# Impacts of *ACE* insertion/deletion variant on cardiometabolic risk factors, premature coronary artery disease, and severity of coronary lesions

**DOI:** 10.1038/s41598-024-64003-w

**Published:** 2024-06-07

**Authors:** Zhi Luo

**Affiliations:** Department of Cardiology, Suining Central Hospital, Suining, 629000 Sichuan China

**Keywords:** Angiotensin-converting enzyme, Cardiometabolic risk factors, Premature coronary artery disease, Multiple vessel lesions, Genetics, Molecular biology, Biomarkers, Cardiology, Diseases, Medical research, Risk factors

## Abstract

Angiotensin-converting enzyme (ACE) is closely related to cardiometabolic risk factors and atherosclerosis. This study aims to investigate whether the insertion/deletion (I/D) variant of *ACE* gene impacts cardiometabolic risk factors, premature coronary artery disease (PCAD), and severity of coronary lesions. PubMed, Cochrane Library, Central, CINAHL, and ClinicalTrials.gov were searched until December 22, 2023. 94,270 individuals were included for the analysis. Carriers of DD genotype had higher levels of triglycerides (TG), total cholesterol (TC), low-density lipoprotein cholesterol (LDL-C), systolic blood pressure (SBP), diastolic blood pressure (DBP), body mass index (BMI), and waist circumference (WC) than carriers of II or ID genotypes. In addition, carriers of DD genotype were at high risk of PCAD and multiple vessel lesions. The impacts of *ACE* I/D variant on lipid levels were significant in American individuals but stronger in male individuals. In contrast, the impacts of *ACE* I/D variant on PCAD and severity of coronary lesions were primarily significant in Caucasian individuals. This study indicates that the *ACE* I/D variant has a slight but significant impact on cardiometabolic risk factors, PCAD, and severity of coronary lesions. Angiotensin-converting enzyme inhibitors (ACEI) may benefit high-risk populations with *ACE* DD genotype to prevent PCAD and multiple vessel lesions.

PROSPERO registration number: CRD42023426732

## Introduction

Clinical and experimental studies demonstrated that the activation of the renin-angiotensin system (RAS) is pivotal in coronary artery disease (CAD) pathogenesis^1,2^. Angiotensin-converting enzyme (ACE), a major component of RAS, converts angiotensin I to angiotensin II (a vasoconstrictor) and degrades bradykinin (a vasodilator). Thus, ACE is critical in regulating the vasomotor tone of arteries. However, chronic exposure to high circulating and tissue levels of ACE predisposes to vascular wall thickening and atherosclerosis^3^.

The human *ACE* gene is located on chromosome 17q23.3 and contains 26 exons and 25 introns. The insertion/deletion variant of the *ACE* gene is characterized by the absence (deletion, D) rather than the presence (insertion, I) of a 287-base pair Alu repeat sequence within intron 16^4^, which results in 3 genotypes: II, ID, and DD. The DD and II genotypes encode high and low activity of ACE^5^, respectively. Individuals of the DD genotype had twofold higher serum ACE levels^6^ than individuals of the II genotype.

Serum ACE levels are closely related to cardiometabolic parameters. For instance, high levels of ACE were associated with high levels of lipid, fasting plasma glucose (FPG), and blood pressure^7^. Using angiotensin-converting enzyme inhibitors (ACEI) improved cardiometabolic parameters (i.e. lipid, FPG, and blood pressure)^8–10^, and attenuated hypercholesterolemia-induced atherosclerotic lesions in rabbits^11,12^ and minipigs^13^. In addition, knockout of the *ACE* gene^14^ inhibited atherosclerosis development in mice subjected to a high-cholesterol diet.

A series of studies^5,6,15^ revealed that serum ACE levels are determined by the *ACE* I/D variant. Since high ACE levels were related to dyslipidemia, dysglycemia, and hypertension^7–14^, it is tempting to speculate that the *ACE* I/D variant may induce dyslipidemia, dysglycemia, and hypertension. According to the newest management strategies for premature coronary artery disease (PCAD)^16^, severe dyslipidemia, diabetes with diabetes-specific risk-enhancing factors, hypertension, or multiple other risk-enhancing factors [e.g. body mass index (BMI) and waist circumference (WC)] were recognized as the primary risk factors for PCAD. In addition to the risk factors in PCAD patients (e.g. dyslipidemia, dysglycemia, and hypertension)^16^ were replicated in those patients with multiple vessel lesions^17^. It is tempting to speculate that the I/D variant of the *ACE* gene may influence the risk of PCAD and the severity of coronary lesions. Here, we conducted this study to investigate this hypothesis.

PCAD is defined as the first onset of CAD in males less than 55 and females less than 65 years of age^18^. Elevated fibrinogen levels were found to be associated with a greater extent of coronary atherosclerosis in women with PCAD but not in men^19^. It indicates that the magnitude of the pathological pathways contributing to PCAD differs in women and men^19^. Despite the already known differences^19^, we lack sufficiently high-quality studies to assess the differences in PCAD formation and progression in different sexes. Here, this study was conducted to clarify this point. Some previous studies have already investigated the impact of the *ACE* I/D variant on PCAD. For instance, Abd El-Aziz et al.^20^ and Berdeli et al.^21^ found that the DD genotype increased the risk of PCAD in the Egyptian and Turkish populations. Niemiec et al.^22^ found that the DD genotype was more frequent in patients with stenoses in at least four coronary vessels or critical arterial occlusions. While Kretowski et al.^23^ reported that patients with type 1 diabetes who had the TT-ID-AA/AC genotype were at high risk of PCAD.

## Results

### Study selection and characteristics

**Figure 1:**
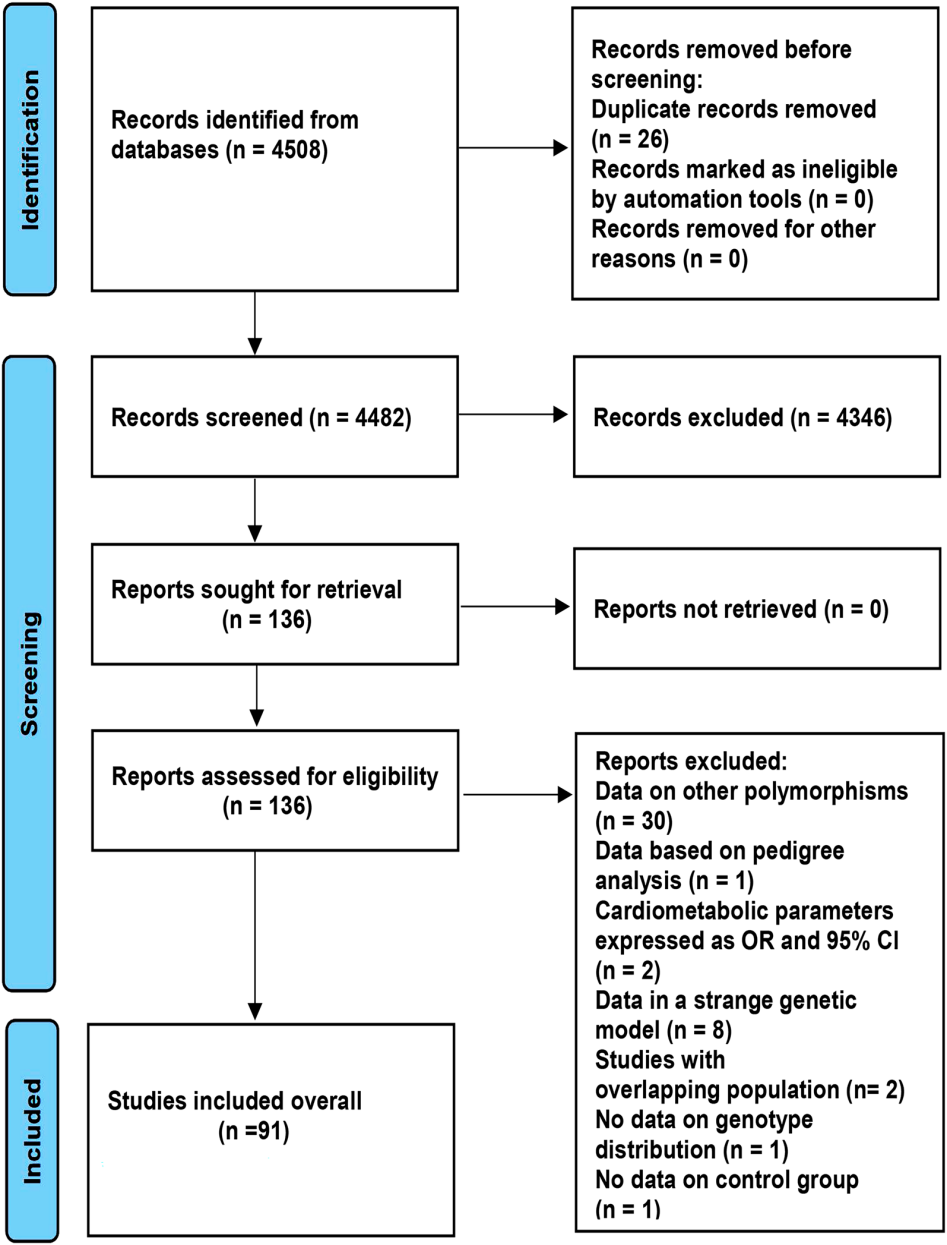
Flow diagram of the studies selection process.

The details of the study selection are summarized in Fig. 1. The present study included 91 studies in a total of 94,270 individuals (Table S1), including 3195 patients with PCAD, and 2395 patients with multiple vessel lesions (Table S1).

### Impact of the *ACE* I/D variant on lipid levels

**Figure 2:**
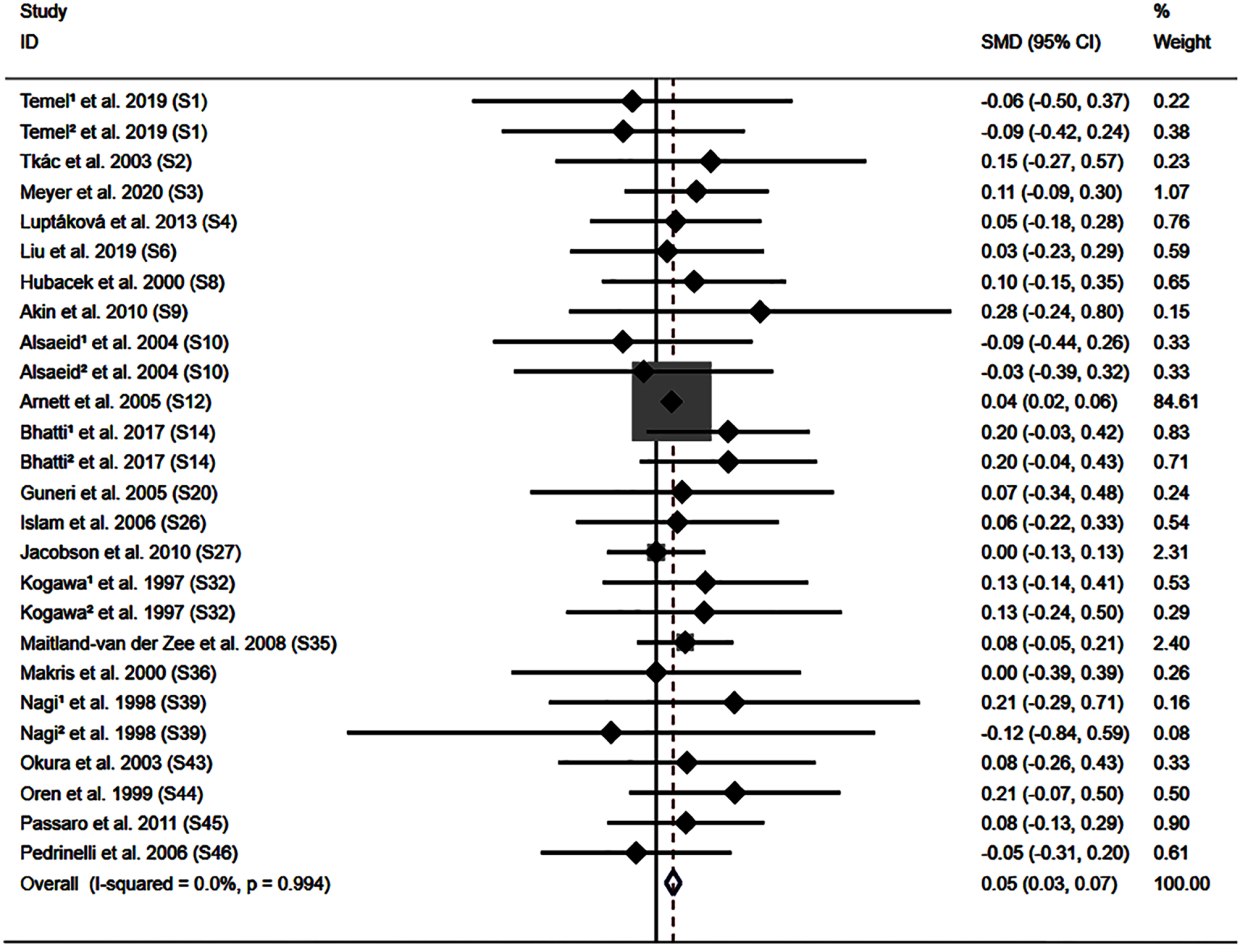
Forest plot of the meta-analysis between ACE DD genotype and low-density lipoprotein cholesterol levels.

All the results stated below were the data that excluded heterogeneity. The DD genotype was linked to higher levels of triglycerides (TG) (Fig. S1), total cholesterol (TC) (Fig. S2), and low-density lipoprotein cholesterol (LDL-C) (Fig. 2). Subgroup analysis by race indicated that the impacts of the *ACE* I/D variant on TG, TC, LDL-C, and high-density lipoprotein cholesterol (HDL-C) levels were consistently significant in American individuals (Table 1). In addition, subgroup analysis by sex indicated that the impacts of the *ACE* I/D variant on TC and LDL-C levels were significant in male individuals (Table 1).

**Table 1:** Meta-analysis of the *ACE* I/D variant with cardiometabolic risk factors.

Groups or subgroups	Subjects	P_H_	SMD (95% CI)	P_SMD_
TG				
All	11,634	0.98	0.07 (0.01–0.13)	0.02
Race				
Caucasian	2780	0.99	0.04 (− 0.04–0.12)	0.38
Asian	6009	0.33	0.11 (− 0.01–0.23)	0.07
American	1117	0.81	0.23 (0.02–0.43)	0.03
Other ethnicities	1728	0.99	0.03 (− 0.09–0.16)	0.56
Sex				
Male	3782	0.98	0.01 (− 0.10–0.13)	0.79
Female	2395	0.87	0.15 (− 0.05–0.35)	0.15
Health status				
CAD	1706	0.71	0.07 (− 0.04–0.18)	0.19
T2DM	1337	0.12	0.09 (− 0.09–0.26)	0.34
Hypertension	342	0.63	-0.22 (− 0.52–0.09)	0.17
CVD	700	0.55	0.02 (− 0.16–0.20)	0.83
Healthy individuals	7103	0.97	0.06 (− 0.02–0.14)	0.15
TC				
All	74,028	0.48	0.04 (0.02–0.06)	< 0.001
Race				
Caucasian	13,361	0.27	0.03 (− 0.01–0.07)	0.18
Asian	9594	0.82	0.08 (− 0.01–0.17)	0.08
American	48,522	0.85	0.04 (0.02–0.06)	< 0.01
Other ethnicities	2551	0.15	0.06 (− 0.02–0.14)	0.15
Sex				
Male	3782	0.84	0.12 (0.00–0.25)	0.05
Female	2500	0.22	0.03 (− 0.06–0.11)	0.53
Health status				
CAD	2488	0.83	0.11 (0.03–0.19)	0.01
T2DM	2394	0.91	0.06 (− 0.11–0.24)	0.45
Hypertension	13,312	0.58	− 0.01 (− 0.05–0.03)	0.70
CVD	38,744	0.09	0.02 (− 0.00–0.05)	0.10
Healthy individuals	15,867	0.16	0.02 (− 0.02–0.06)	0.48
LDL-C				
All	57,155	0.99	0.05 (0.03–0.07)	< 0.001
Race				
Caucasian	3639	0.98	0.04 (− 0.03–0.12)	0.24
Asian	3133	0.95	0.09 (− 0.06–0.24)	0.26
American	47,823	0.13	0.03 (0.00–0.05)	0.02
Other ethnicities	2560	0.83	0.09 (0.01–0.17)	0.02
Sex				
Male	1421	0.50	0.30 (0.15–0.45)	0.03
Female	419	0.66	0.20 (− 0.12–0.52)	0.82
Health status				
CAD	2154	0.55	0.03 (− 0.06–0.11)	0.53
T2DM	2015	0.30	0.02 (− 0.04–0.08)	0.49
Hypertension	9809	0.63	0.03 (− 0.02–0.08)	0.21
CVD	38,044	0.66	0.05 (0.03–0.08)	< 0.001
Healthy individuals	3022	0.88	0.09 (− 0.01–0.18)	0.08
HDL-C				
All	71,998	0.29	0.01 (− 0.00–0.03)	0.15
Race				
Caucasian	12,984	0.77	0.01 (− 0.03–0.05)	0.54
Asian	7364	0.45	− 0.04 (− 0.11–0.03)	0.22
American	48,522	0.78	0.02 (0.00–0.05)	0.02
Other ethnicities	3128	0.10	− 0.08 (− 0.15–0.01)	0.03
Sex				
Male	3697	0.44	− 0.06 (− 0.15–0.02)	0.15
Female	2395	0.96	− 0.02 (− 0.13–0.09)	0.71
Health status				
CAD	2818	0.85	0.02 (− 0.05–0.10)	0.54
T2DM	5520	0.33	− 0.03 (− 0.09–0.03)	0.35
Hypertension	9809	0.63	0.03 (− 0.02–0.08)	0.21
CVD	38,639	0.66	0.05 (0.03–0.08)	< 0.001
Healthy individuals	13,989	0.14	− 0.02 (− 0.06–0.02)	0.29
Fasting plasma glucose				
All	5016	0.23	0.05 (− 0.03–0.12)	0.24
Race				
Caucasian	786	0.37	0.06 (− 0.08–0.21)	0.40
Asian	3957	0.09	0.02 (− 0.07–0.11)	0.67
Other ethnicities	273	0.30	0.19 (− 0.07–0.45)	0.16
Sex				
Male	2273	0.40	− 0.03 (− 0.15–0.08)	0.56
Female	2271	0.63	0.07 (− 0.04–0.18)	0.22
Health status				
Healthy individuals	4572	0.41	0.02 (− 0.06–0.10)	0.60
Systolic blood pressure				
All	58,106	0.78	0.07 (0.04–0.11)	< 0.001
Race				
Caucasian	11,387	0.88	0.05 (0.01–0.09)	0.01
Asian	7563	0.26	0.08 (− 0.02–0.18)	0.12
American	37,996	0.97	0.05 (0.03–0.08)	< 0.001
Other ethnicities	1160	0.99	0.17 (0.05–0.29)	0.01
Sex				
Male	2654	0.28	0.15 (0.05–0.25)	< 0.01
Female	2376	0.93	0.03 (− 0.08–0.15)	0.58
Health status				
CAD	562	0.50	0.26 (0.01–0.51)	0.04
T2DM	3971	0.91	0.07 (0.00–0.14)	0.05
Hypertension	342	0.31	0.06 (− 0.25–0.36)	0.72
CVD	38,044	0.83	0.05 (0.03–0.08)	< 0.001
Healthy individuals	14,761	0.40	0.07 (0.03–0.11)	< 0.001
Diastolic blood pressure				
All	57,975	0.21	0.09 (0.07–0.11)	< 0.001
Race				
Caucasian	11,313	1.00	0.03 (− 0.01–0.07)	0.20
Asian	7563	0.10	0.03 (− 0.02–0.07)	0.23
American	–	–	–	–
Other ethnicities	1160	0.07	0.13 (0.01–0.25)	0.04
Sex				
Male	2689	0.81	0.14 (0.04–0.24)	0.01
Female	2376	0.75	0.06 (− 0.05–0.18)	0.28
Health status				
T2DM	3805	0.99	0.02 (− 0.04–0.09)	0.48
Hypertension	342	0.48	0.12 (− 0.19–0.43)	0.43
CVD	38,044	0.90	0.04 (0.02–0.06)	< 0.01
Healthy individuals	14,761	0.56	0.06 (0.02–0.10)	0.01
Body mass index				
All	6637	0.71	0.08 (0.02–0.14)	0.01
Race				
Caucasian	1020	0.49	0.03 (− 0.09–0.16)	0.59
Asian	4545	0.33	0.07 (− 0.01–0.16)	0.08
Other ethnicities	1015	0.86	0.18 (0.02–0.34)	0.03
Sex				
Male	2435	0.66	0.05 (− 0.06–0.16)	0.38
Female	2271	0.19	0.10 (− 0.02–0.21)	0.10
Health status				
CAD	424	0.98	0.18 (− 0.05–0.42)	0.12
T2DM	413	0.60	0.10 (− 0.15–0.35)	0.44
Hypertension	342	0.86	0.27 (− 0.04–0.58)	0.09
Healthy individuals	4800	0.78	0.09 (0.01–0.17)	0.02
Waist circumference				
All	1342	0.12	0.28 (0.13–0.43)	< 0.001
Race				
Other ethnicities	1163	0.31	0.35 (0.13–0.58)	< 0.01
Sex				
Female	309	0.38	0.20 (− 0.06–0.45)	0.14
Health status				
MetS	113	0.21	0.19 (− 0.20–0.59)	0.34
Obesity	641	0.31	0.35 (0.13–0.58)	< 0.01
Healthy individuals	213	0.47	0.31 (0.02–0.61)	0.04

*ACE* angiotensin converting enzyme gene, *SMD* standardized mean difference, *95% CI* 95% confidence interval, *P*_H_ P for heterogeneity, *CAD* coronary artery disease, *T2DM* type 2 diabetes mellitus, *Mets* metabolic syndrome, *CVD* cardiovascular disease, *TG* triglycerides, *TC* total cholesterol, *LDL*-*C* low-density lipoprotein cholesterol, *HDL*-*C* high-density lipoprotein cholesterol.

### Impacts of the *ACE* variant on other cardiometabolic risk factors

**Figure 3:**
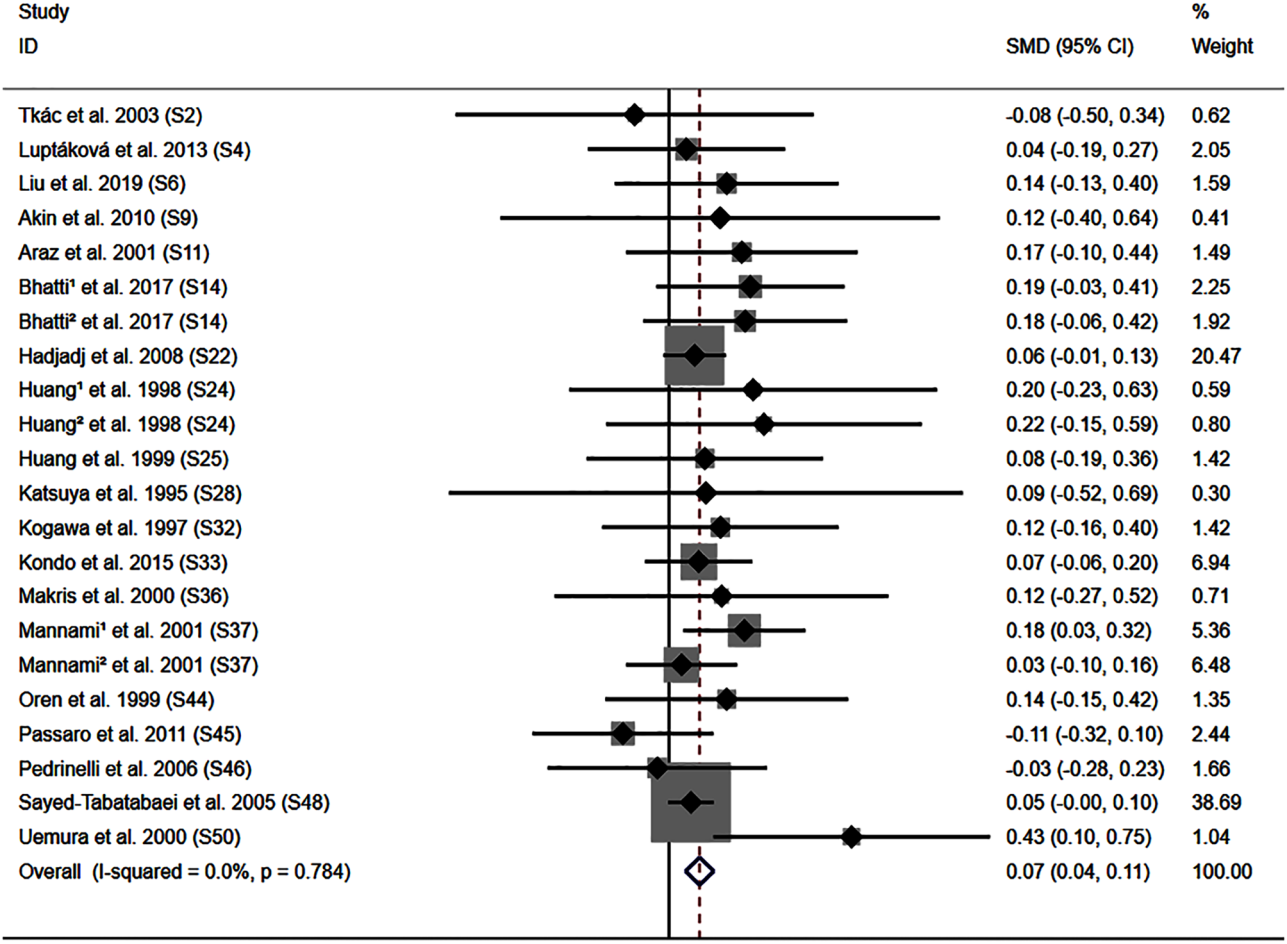
Forest plot of the meta-analysis between ACE DD genotype and systolic blood pressure levels.

The DD genotype was associated with elevated systolic blood pressure (SBP) (Fig. 3), diastolic blood pressure (DBP) (Fig. S3), BMI (Fig. S4), and WC (Fig. S5). Subgroup analysis indicated that the impact of the *ACE* I/D variant on SBP was significant in Caucasian individuals, American individuals, male individuals, patients with CAD, and patients with type 2 diabetes mellitus (T2DM) (Table 1). In contrast, the impact of the *ACE* I/D variant on DBP was significant in male individuals and patients with cardiovascular disease (CVD) (Table 1). In addition, the impacts of the *ACE* I/D variant on BMI and WC were significant in other ethnicities individuals (Table 1). However, the impact of the *ACE* I/D variant on FPG levels did not show statistically significant (Table 1).

### Impact of the *ACE* I/D variant on the risk of PCAD

**Figure 4:**
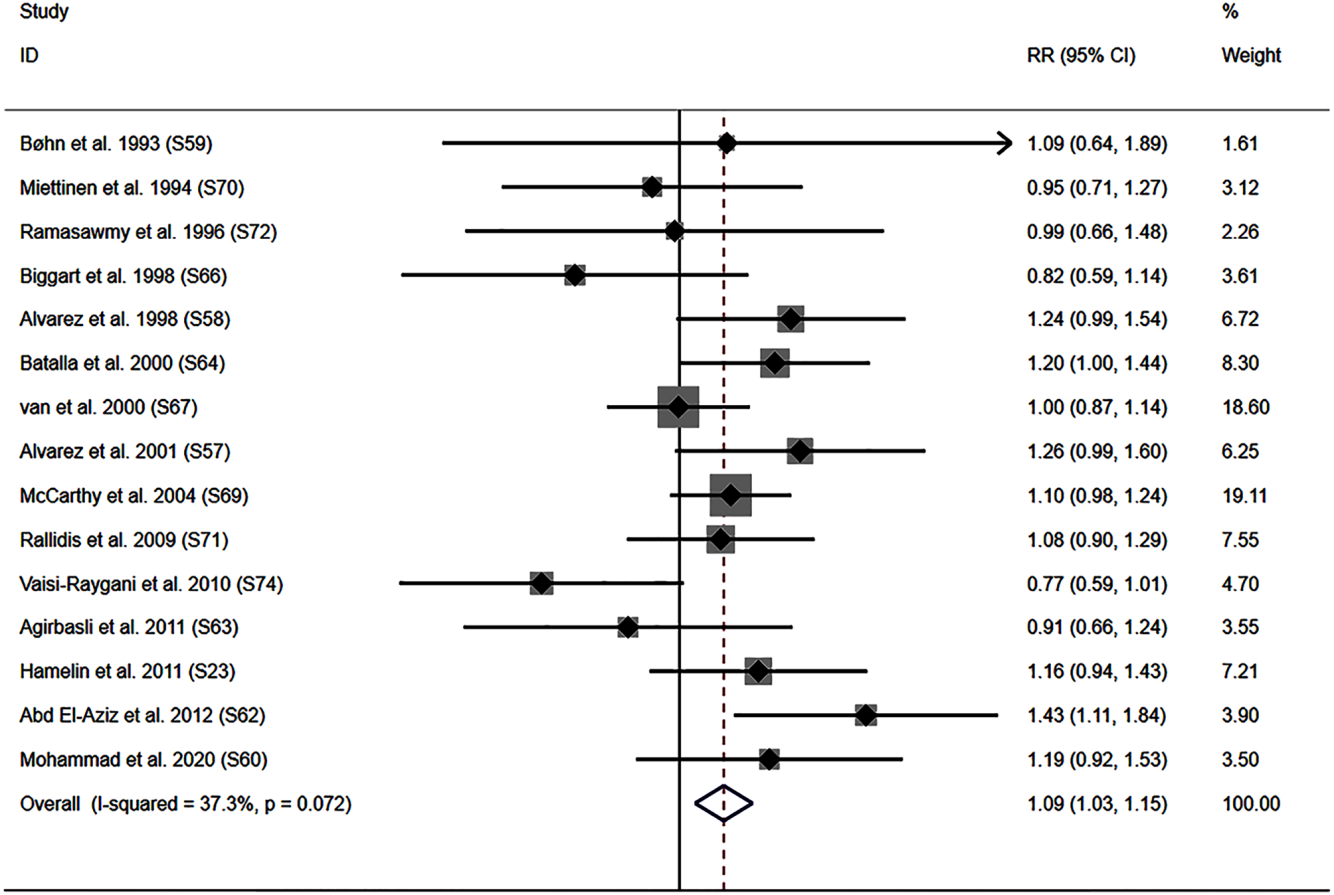
Forest plot of the meta-analysis between ACE DD genotype and premature coronary artery disease risk.

The DD genotype increased the risk of PCAD compared with II or ID genotypes (Fig. 4). Subgroup analysis indicated that the impact of the *ACE* I/D variant on PCAD risk was primarily significant in Caucasian and male individuals (Table 2).

**Table 2:** Meta-analysis of the *ACE* I/D variant with premature coronary artery disease.

Groups or subgroups	Subjects	P_H_	RR (95% CI)	P_RR_
Allelic model (D vs. I)				
All	11,116	0.18	1.05 (1.01–1.10)	0.03
Caucasian	9560	0.52	1.05 (1.00–1.10)	0.04
Other ethnicities	1556	0.03	1.06 (0.94–1.20)	0.38
Male	3118	0.32	1.13 (1.04–1.23)	0.01
Additive model (DD vs. II)				
All	3250	0.15	1.04 (0.95–1.14)	0.40
Caucasian	2817	0.30	1.01 (0.96–1.07)	0.59
Other ethnicities	433	0.11	1.06 (0.93–1.21)	0.36
Male	1057	0.05	1.05 (0.96–1.14)	0.28
Heterozygote model (ID vs. II)				
All	4353	0.06	1.02 (0.98–1.06)	0.26
Caucasian	3471	0.09	1.04 (1.00–1.09)	0.05
Other ethnicities	882	0.35	0.94 (0.86–1.03)	0.17
Male	1222	0.53	0.98 (0.91–1.06)	0.65
Dominant model (ID + DD vs. II)				
All	3660	0.11	1.03 (1.00–1.06)	0.04
Caucasian	2487	0.41	1.01 (0.97–1.04)	0.68
Other ethnicities	1173	0.17	1.07 (1.02–1.13)	< 0.01
Male	1545	0.29	0.98 (0.94–1.03)	0.41
Recessive model (DD vs. II + ID)				
All	3702	0.07	1.09 (1.03–1.15)	< 0.01
Caucasian	3219	0.61	1.12 (1.04–1.21)	< 0.01
Other ethnicities	483	0.50	1.08 (0.90–1.29)	0.42
Male	970	0.91	1.20 (1.06–1.36)	< 0.01
Overdominant model (ID vs. II + DD)				
All	3692	0.29	1.09 (1.05–1.14)	< 0.001
Caucasian	2918	0.07	1.10 (1.03–1.16)	< 0.01
Other ethnicities	774	0.58	1.19 (1.06–1.34)	< 0.01
Male	1057	0.14	1.04 (0.94–1.15)	0.40

*ACE* angiotensin converting enzyme gene, *RR* risk ratios, *95% CI* 95% confidence interval, *P*_H_ *P* for heterogeneity.

### Impact of the *ACE* I/D variant on the severity of coronary lesions

**Figure 5:**
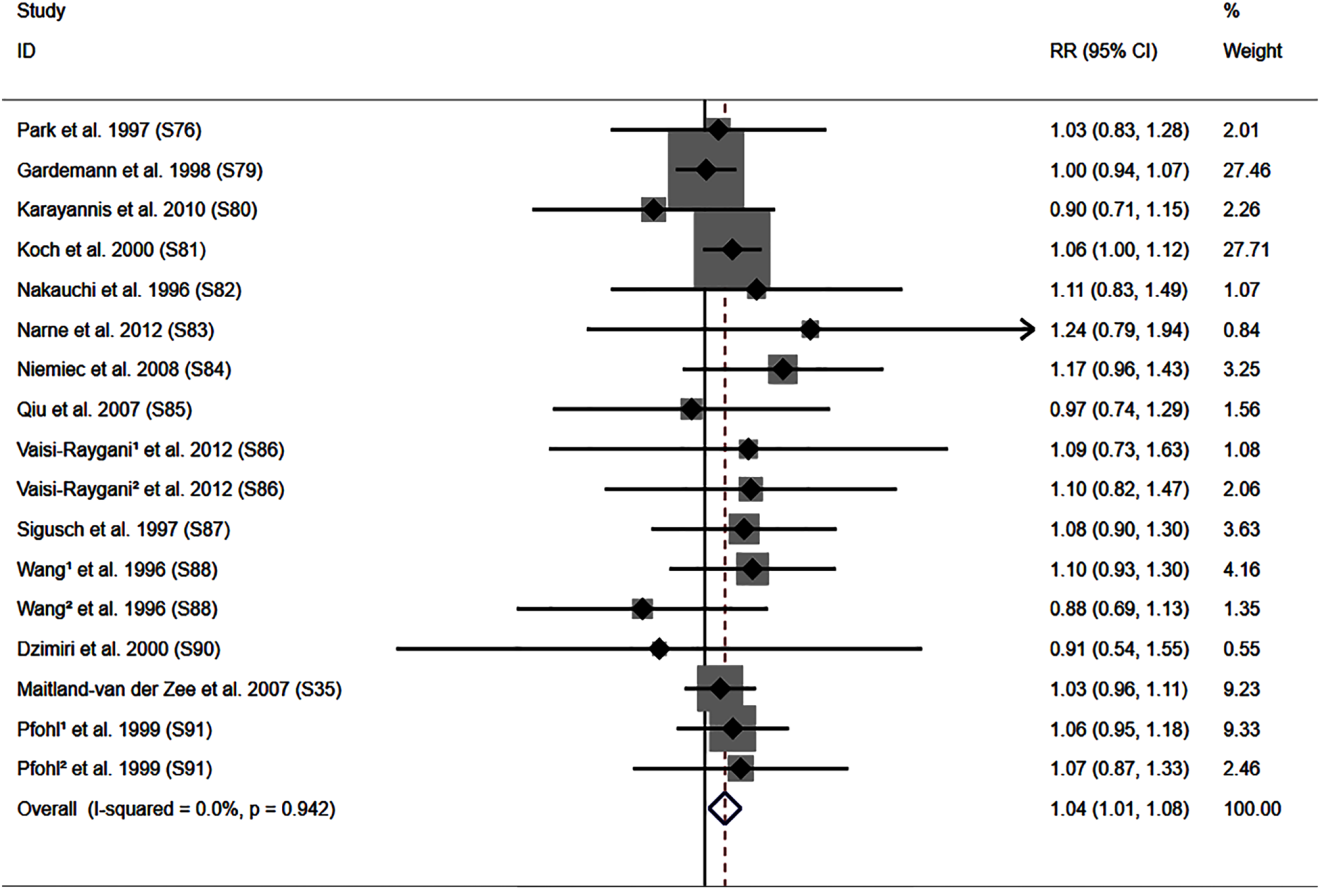
Forest plot of the meta-analysis between ACE DD genotype and severity of coronary lesions.

The DD genotype increased the risk of multiple vessel lesions compared with II or ID genotypes (Fig. 5). Subgroup analysis indicated that the impact of the *ACE* I/D variant on multiple vessel lesions was significant in Caucasian and other ethnicities individuals (Table 3).

**Table 3:** Meta-analysis of the *ACE* I/D variant with severity of coronary lesions.

Groups or subgroups	Subjects	P_H_	RR (95% CI)	P_RR_
Allelic model (D vs. I)				
All	15,824	0.79	1.07 (1.03–1.11)	< 0.001
Caucasian	12,006	0.50	1.07 (1.02–1.12)	< 0.01
Asian	1762	0.10	1.06 (0.98–1.14)	0.11
Other ethnicities	2056	0.93	1.12 (1.00–1.26)	0.05
Male	2254	0.96	1.06 (0.96–1.17)	0.24
Female	560	0.22	0.96 (0.78–1.17)	0.69
Additive model (DD vs. II)				
All	3907	0.37	1.12 (1.05–1.20)	< 0.01
Caucasian	3891	0.51	1.12 (1.03–1.22)	0.01
Asian	730	0.14	1.07 (0.96–1.19)	0.18
Other ethnicities	602	0.85	1.25 (0.98–1.60)	0.07
Male	622	0.97	1.11 (0.91–1.37)	0.31
Female	150	0.28	0.90 (0.60–1.37)	0.63
Heterozygote model (ID vs. II)				
All	5286	0.33	1.08 (1.01–1.16)	0.03
Caucasian	3694	0.56	1.06 (0.98–1.15)	0.14
Asian	939	0.08	1.08 (0.95–1.23)	0.23
Other ethnicities	653	0.36	1.22 (0.95–1.58)	0.12
Male	752	0.57	0.99 (0.80–1.23)	0.94
Female	188	0.82	0.83 (0.53–1.29)	0.40
Dominant model (ID + DD vs. II)				
All	5883	0.40	1.05 (1.02–1.08)	< 0.01
Caucasian	4421	0.41	1.03 (1.01–1.07)	0.02
Asian	661	0.20	1.08 (0.97–1.2.0)	0.14
Other ethnicities	801	0.58	1.07 (0.99–1.15)	0.06
Male	880	0.85	1.01 (0.95–1.08)	0.65
Female	222	0.51	0.95 (0.83–1.09)	0.46
Recessive model (DD vs. II + ID)				
All	5286	0.94	1.04 (1.01–1.08)	0.01
Caucasian	3694	0.71	1.08 (1.01–1.16)	0.02
Asian	939	0.53	1.10 (0.86–1.40)	0.43
Other ethnicities	653	0.90	1.13 (0.94–1.36)	0.19
Male	752	0.70	1.13 (0.95–1.33)	0.17
Female	188	0.26	1.01 (0.70–1.47)	0.94
Overdominant model (ID vs. II + DD)				
All	5223	0.93	0.99 (0.93–1.05)	0.75
Caucasian	3891	0.82	1.01 (0.93–1.08)	0.88
Asian	730	0.72	0.93 (0.79–1.09)	0.38
Other ethnicities	989	0.65	0.97 (0.83–1.15)	0.75
Male	622	0.45	1.06 (0.93–1.23)	0.36
Female	150	0.62	1.10 (0.83–1.46)	0.49

*ACE* angiotensin converting enzyme gene, *RR* risk ratios, *95% CI* 95% confidence interval, *P*_H_ *P* for heterogeneity.

### Publication bias test

Begg funnel plot was used to evaluate publication bias among the included studies. This meta-analysis had no publication bias, which was confirmed by the Egger linear regression test.

## Discussion

The present study indicated that the DD genotype of the *ACE* I/D variant increased the risk of PCAD (Table 2) and multiple vessel lesions (Table 3), as well as elevated TG, TC, LDL-C, SBP, DBP, BMI, and WC (Table 1). Since dyslipidemia, hypertension, high BMI, and large WC were considered the primary risk factors for PCAD and multiple vessel lesions^16,17^, it indicated that the increased risk of PCAD (Table 2) and multiple vessel lesions (Table 3) associated with DD genotype was attributed, at least partly, by the impacts of the *ACE* I/D variant on TG, TC, LDL-C, SBP, DBP, BMI, and WC (Table 1).

Subgroup analyses by sex revealed that the impact of the *ACE* I/D variant on PCAD was significant in male individuals (Table 2). Since the impacts of the *ACE* I/D variant on LDL-C, TC, SBP, and DBP were significant in male individuals (Table 1), it indicated that the impact of the *ACE* I/D variant on PCAD in male individuals (Table 2) was at least partly mediated by the elevated LDL-C, TC, SBP, and DBP (Table 1). In addition, subgroup analyses by race showed that the impacts of the *ACE* I/D variant on PCAD and multiple vessel lesions were significant in Caucasian individuals (Tables 2, 3). Since the impact of the *ACE* I/D variant on SBP was significant in Caucasian individuals (Table 1), it indicated that the impacts of the *ACE* I/D variant on PCAD and CAD severity in Caucasian individuals (Tables 2, 3) were partially mediated by the elevated SBP (Table 1).

In this study, the prevalence of II genotype in Caucasian individuals, American individuals, Asian individuals, and other ethnicities individuals was 0.40 (range from 0.08 to 0.72), 0.43 (range from 0.22 to 0.64), 0.50 (range from 0.22 to 0.78), and 0.535 (range from 0.22 to 0.85), respectively. In contrast, the prevalence of DD genotype in Caucasian individuals, American individuals, Asian individuals, and other ethnicities individuals was 0.325 (range from 0.15 to 0.50), 0.275 (range from 0.18 to 0.37), 0.23 (range from 0 to 0.46), and 0.17 (range from 0.04 to 0.30), respectively. In addition, the prevalence of ID genotype in Caucasian individuals, American individuals, Asian individuals, and other ethnicities individuals was 0.275 (range from 0.22 to 0.33), 0.295 (range from 0.18 to 0.41), 0.27 (range from 0.205 to 0.335), and 0.295 (range from 0.15 to 0.44), respectively.

The impacts of the *ACE* I/D variant on PCAD and the severity of coronary lesions were primarily significant in Caucasian individuals, but not in Asian individuals and other ethnicities individuals (Tables 2, 3). One particular reason could be proposed to interpret this phenomenon. That is, Caucasian individuals had a higher carrying rate of DD than Asian and other ethnicities individuals (Caucasian individuals vs. Asian individuals vs. Other ethnicities individuals = 0.325 vs. 0.23 vs. 0.17). Similarly, since the prevalence rate of DD was relatively higher in American individuals than in Asian individuals and other ethnicities individuals (American individuals vs. Asian individuals vs. other ethnicities individuals = 0.275 vs. 0.23 vs. 0.17), it is plausible to observe that the impacts of the *ACE* I/D variant on cardiometabolic parameters (Table 1) were primarily significant in American individuals.

According to the 2018 American College of Cardiology (ACC)/American Heart Association (AHA)^24^, the 2019 European Society of Cardiology (ESC)/European Atherosclerosis Society (EAS)^25^, and the Adult Treatment Panel III (ATP III) cholesterol guidelines^26^, LDL-C was considered the major cause of CAD and treated as the primary target for therapy, while other lipids were used as the secondary or supplementary therapeutic targets. In this study, the strongest impact of the *ACE* I/D variant on LDL-C levels was detected in male individuals (SMD = 0.30, 95% CI 0.15 to 0.45, *P* = 0.03) (Table 1). It indicated that male individuals with DD were at a very high risk of CAD. In addition, the impact of the *ACE* I/D variant on LDL-C levels was also significant in American individuals (Table 1), suggesting that American individuals with DD were at high risk of CAD.

A small sample case–control study conducted by Winkelmann and colleagues^27^ included 209 male patients with CAD and 92 male controls and found that the *ACE* I/D variant was not associated with an increased risk for CAD or myocardial infarction (MI). In contrast, a moderate-scale case–control study conducted by Cambien and colleagues^28^ included 610 patients with MI and 733 controls and found that the *ACE* I/D variant was a potent risk factor for CAD in subjects formerly considered to be at low risk according to common criteria. In addition, Miao et al.^29^, Zhou et al.^30^, and Zhang et al.^31^ respectively conducted a meta-analysis (sample size: 1241 cases and 3452 controls for Miao et al. 5215 cases and 4782 controls for Zhou et al. 5619 cases and 4865 controls for Zhang et al.) to investigate the association between *ACE* I/D variant and the risk of CAD. Intriguingly, all three mete-analyses demonstrated that there was a significant association between *ACE* I/D variant and CAD [odds ratio (OR) = 1.92 for Miao et al., OR range from 1.19 to 2.40 for Zhou et al., OR = 1.95 for Zhang et al.]. In line with previous clinical trials^21,22,32–40^. Taken together, the correlation between the *ACE* I/D variant and CAD appears to be related to the sample size of studies. In which, studies with small samples are unlikely to detect the true impact of the *ACE* I/D variant on CAD, whilst studies with relatively larger samples may detect the true association between the *ACE* I/D variant and CAD due to adequate statistical power.

The present meta-analysis included 3195 patients with PCAD and 2395 patients with multiple vessel lesions and found that the DD genotype significantly increased the risk of PCAD and multiple vessel lesions by 9% and 4%, respectively, suggesting that the *ACE* I/D variant was slightly but significantly associated with PCAD and multiple vessel lesions (Tables 2, 3). In line with these findings, this meta-analysis also indicated that the DD genotype significantly increased TG, TC, LDL-C, SBP, DBP, BMI, and WC (Table 1). It indicated that the impacts of the *ACE* I/D variant on PCAD and the severity of coronary lesions (Tables 2, 3) were at least partly mediated by the impacts of the *ACE* I/D variant on cardiometabolic risk factors (Table 1).

The impacts of the *ACE* I/D variant on cardiometabolic risk factors, PCAD, and severity of coronary lesions (Tables 1, 2 and 3) were essentially attributed to high levels of ACE^7–14,16,17^. Since ACEI/angiotensin receptor blockers (ARBs) were prominent inhibitors of ACE that had been widely used in clinical practice^41^, it is tempting to hypothesize that preventive use of ACEI/ARBs may benefit high-risk populations with the DD genotype to prevent cardiometabolic disorder, PCAD, and multiple vessel lesions. Further randomized controlled trials (RCTs) are needed to verify this hypothesis and detail the dose, frequency, and duration of administration. Since the DD genotype was closely linked to cardiometabolic disorder (Table 1), PCAD (Table 2), and multiple vessel lesions (Table 3), the genetic screening of the *ACE* I/D variant may benefit the early prevention or control of PCAD and multiple vessel lesions.

The connection between genotype and phenotype is intricate. For instance, the genotypes may generate complex clinical phenotypes, which can be further modified by external factors (e.g. environmental factors and lifestyles). In other words, the clinical phenotypes of individual organisms are jointly determined by multiple factors (e.g. genetic factors, environmental factors, and personal lifestyles). Additionally, there are often many genotypes that produce the same phenotype, adding a layer of complexity in establishing valid genotype–phenotype relationships^42^. Therefore, prediction of trait heritability via genotype–phenotype association remains a major challenge in modern genetics. How to reasonably regulate the trait inheritance association with heterogeneous genotype–phenotype is becoming an important field of biological research and application, and it is also the prerequisite and foundation to execute gene editing and gene therapy.

The present meta-analysis has several strengths (1) all results are recalculated after excluding the studies with heterogeneity (Table 1), which advances the preciseness of conclusions drawn in this paper; (2) the conclusions will no doubt benefit clinicians to make the optimum management strategies for high-risk population with the DD genotype to prevent PCAD or multiple vessel lesions; (3) genetic screening of the DD genotype is critical for the high-risk population to prevent PCAD or multiple vessel lesions. In addition, several limitations should be noted (1) there is no information regarding the moment of measurement (on treatment or without treatment); (2) CAD is a multifactorial disease, and the major risk factors include genetic factors, environmental influences, and personal lifestyles. To explain the role of specific mutation (e.g. *ACE* I/D variant), all relevant risk factors (i.e. genetic factors, environmental influences, and personal lifestyles) should be explored. However, the impacts of environmental factors and personal lifestyles associated with the *ACE* I/D variant on PCAD and CAD severity, as well as the interactions of the *ACE* I/D variant with environmental factors and personal lifestyles on PCAD and CAD severity have yet to be investigated in the present meta-analysis due to the lack of original data from the included studies. In other words, more precise results could have been gained if more detailed individual data were available or the stratification analyses based on environmental factors and personal lifestyles, such as diet, exercise, smoking, alcohol consumption, etc., were performed; (3) there was no study investigated the impact of *ACE* I/D variant on PCAD in Asian individuals, American individuals, and female individuals (Table 2). More comprehensive and diversified results would be available if more studies on specific populations (e.g. Asian individuals, American individuals, and female individuals) were carried out.

## Materials and methods

The current meta-analysis follows the Preferred Reporting Items for Systematic Reviews and Meta-analyses (see Table S2 for more details)^43^.

### Literature search

A comprehensive literature search was performed from January 18, 2021 to December 22, 2023 using PubMed, Cochrane Library, Central, CINAHL, and ClinicalTrials.gov. The following keywords were used in the search: [“angiotensin-converting enzyme,” “renin-angiotensin system”] AND [“variant,” “polymorphism,” “mutation,” “variation,” “mutant,” “SNP”] OR [“single nucleotide polymorphism”] AND [“lipid,” “circulating lipids,” “blood lipids,” “plasma lipids,” “serum lipids,” “lipid profile”] OR [“triglycerides,” “total cholesterol,” “low-density lipoprotein cholesterol,” “high-density lipoprotein cholesterol”] OR [“fasting plasma glucose,” “fasting blood glucose”] OR [“blood pressure,” “systolic blood pressure,” “diastolic blood pressure”] OR [“body mass index”] OR [“waist circumference”] OR [“premature coronary artery disease,” “early-onset coronary artery disease”] AND/OR [“coronary artery disease severity,” “triple-vessel lesions,” “multiple-vessel lesions”]. Additionally, the reference lists of all eligible studies were manually retrieved to search for additional literature.

### Inclusion criteria

The inclusion criteria for the impacts of the *ACE* I/D variant on PCAD and CAD severity include (1) case–control design; (2) CAD cases were angiographically defined; (3) studies provided the number of individual genotypes in cases and controls for the *ACE* I/D variant; (4) studies provided the number of individual genotypes in multiple (≥ 3) and non-multiple (< 3) lesions for the *ACE* I/D variant. The inclusion criteria for the impacts of the *ACE* I/D variant on cardiometabolic risk factors include (1) studies investigated the association of *ACE* I/D variant with lipid [including four lipid parameters, i.e. TG, TC, LDL-C, and HDL-C, rather than other lipoproteins, e.g. apolipoprotein B (APOB), apolipoprotein E (APOE), and apolipoprotein A1 (APOA1), etc.; expressed as mean with standard deviation (SD) or standard errors (SE), but not other forms, such as, mean with 95% confidence intervals (CI) and median with interquartile range (IQR); data of dyslipidemia and normal lipid levels were available]; (2) studies investigated the association of *ACE* I/D variant with blood pressure (including SBP and DBP; expressed as mean with SD or SE; data of hypertension and normal blood pressure levels were available); (3) studies investigated the association of the *ACE* I/D variant with FPG (rather than postprandial plasma glucose; expressed as mean with SD or SE; data of dysglycemia and normal plasma glucose were available); (4) studies investigated the association of *ACE* I/D variant with BMI [expressed as mean with SD or SE; data of underweight (< 18.5 kg/m^2^), overweight (25–29.9 kg/m^2^), obesity (> 30 kg/m^2^), and normal range (18.5–24.9 kg/m^2^) were available]; (5) studies investigated the association of *ACE* I/D variant with WC (expressed as mean with SD or SE); (6) studies provided the number of individual genotypes for the *ACE* I/D variant; (7) the language of eligible studies was restricted to English. The exclusion criteria of this meta-analysis include (1) studies that were not related to the *ACE* I/D variant; (2) studies that were not related to cardiometabolic risk factors; (3) studies that were not related to PCAD or CAD severity; (4) studies not presenting genotype count in controls, or the genotype distribution of controls deviate from the Hardy–Weinberg equilibrium (HWE); (5) studies having invalid data; (6) studies having incomplete data; (7) pedigree studies; (8) overlapping studies; and (9) abstract, review, case report, meta-analysis, and animal studies.

### Data extraction

The data extraction was conducted by ZL. From each included study, the following was extracted: the last name of the first author; publication time; country, race, sex, age, case and control counts, genotype count, study design, study period, and mean lipid, blood pressure, FPG, BMI, and WC with SD or SE by the genotype of the *ACE* I/D variant (i.e. II, ID, and DD).

### Data analysis

The units of TG, TC, LDL-C, HDL-C, and FPG were converted into mmol/L. The unit of blood pressure was converted into mmHg. The unit of BMI was converted into kg/m^2^. The unit of WC was converted into cm. All extracted data were expressed as mean ± SD. Risk ratios (RR) and corresponding 95% CI were used to evaluate the strength of the *ACE* I/D variant in PCAD and CAD severity. Standardized mean difference (SMD) with 95% CI was used to evaluate the differences in cardiometabolic parameters (i.e. TG, TC, LDL-C, HDL-C, SBP, DBP, FPG, BMI, and WC) between carriers of DD and carriers of II or ID. The pooled RR was performed in the allelic model (*D *vs*. I*), additive model (*DD *vs*. II*), heterozygote model (*ID *vs*. II*), dominant model (*ID* + *DD *vs*. II*), recessive model (*DD *vs*. II* + *ID*), and overdominant model (*ID *vs*. II* + *DD*). Since most publications compared cardiometabolic parameters between carriers of DD and carriers of II or ID, a recessive genetic model (*DD *vs*. II* + *ID*) was adopted to analyze the strength of the *ACE* I/D variant in cardiometabolic parameters. All statistical tests were conducted with the Cochrane Collaboration meta-analysis software (Review Manager 5.4). *P* < 0.05 was recognized as statistically significant.

### Subgroup analysis

Subgroup analysis was performed by race, sex, and health status. The race was divided into Caucasian, Asian, American, and other ethnicities. Healthy status was divided into CAD, T2DM, hypertension, obesity, metabolic syndrome (Mets), CVD, and healthy individuals. In some studies, subjects were divided into multiple subpopulations (e.g. patients with different diseases or individuals from different races, etc.). Each subpopulation was regarded as an independent comparison in this study.

### Evaluation of heterogeneity

Heterogeneity was tested by *I*^2^ statistic and Cochran's χ^2^-based Q statistic. If heterogeneity was significant (*I*^2^ > 50%, *P* ≤ 0.05), the random-effect model (DerSimonian-Laird method) was used to calculate the results^44^. Otherwise, the fixed-effect model (Mantel–Haenszel method) would be adopted (*I*^2^ < 50%, *P* > 0.05). In addition, the Galbraith plot was employed to detect the potential sources of heterogeneity. To eliminate the impact of heterogeneity on the ultimate results, all preliminary results were recalculated after excluding the studies with heterogeneity.

### Publication bias test

The Begg funnel plot and Egger linear test evaluated the probability of publication bias among the included studies^45^.

### The summary statistics of this study

#### Primary results

1. The differences in cardiometabolic parameters (i.e. TG, TC, LDL-C, HDL-C, FPG, SBP, DBP, BMI, and WC) between carriers of DD and carriers of II or ID in an integrated population (i.e. Caucasian, Asian, American, and other ethnicities).
2. The impact of *ACE* I/D variant on PCAD risk in allelic model (*D *vs*. I*), additive model (*DD *vs*. II*), heterozygote model (*ID *vs*. II*), dominant model (*ID* + *DD *vs*. II*), recessive model (*DD *vs*. II* + *ID*), and overdominant model (*ID *vs*. II* + *DD*).
3. The impact of *ACE* I/D variant on CAD severity in allelic model (*D *vs*. I*), additive model (*DD *vs*. II*), heterozygote model (*ID *vs*. II*), dominant model (*ID* + *DD *vs*. II*), recessive model (*DD *vs*. II* + *ID*), and overdominant model (*ID *vs*. II* + *DD*).

#### Secondary results

1. The differences in cardiometabolic parameters (i.e. TG, TC, LDL-C, HDL-C, FPG, SBP, DBP, BMI, and WC) between carriers of DD and carriers of II or ID in specific populations, including Caucasian, Asian, American, and other ethnicities, males, females, CAD patients, T2DM patients, hypertension patients, obesity patients, Mets patients, CVD patients, and healthy individuals.
2. The impact of *ACE* I/D variant on PCAD risk in Caucasians, other ethnicities, and males under different genetic models.
3. The impact of *ACE* I/D variant on CAD severity in Caucasians, Asians, other ethnicities, males, and females under different genetic models.

### Supplementary Information

Supplementary Information.

## Data Availability

The datasets generated during and/or analysed during the current study are available from the corresponding author on reasonable request.
