# Supplementary material for: Impacts of ACE insertion/deletion variant on cardiometabolic risk factors, premature coronary artery disease, and severity of coronary lesions
Source: Sci Rep. 2024 Jun 7;14:13171. doi: 10.1038/s41598-024-64003-w (PMC11161653; doi:10.1038/s41598-024-64003-w)
Supplement: Supplementary file 1 — Supplementary Information. [file 41598_2024_64003_MOESM1_ESM.doc]

**Supplementary Material**

**Impacts of *ACE* insertion/deletion variant on cardiometabolic risk factors, premature coronary artery disease, and severity of coronary lesions**

Zhi Luo

**Supplemental Tables**

*Table S1* **Characteristics of the included studies.**

*Table S2* **Checklist of items to include when reporting a systematic review or meta-analysis.**

**Supplemental Figures**

*Figure S1* **Forest plot of the meta-analysis between *ACE* DD genotype and triglycerides levels.**

*Figure S2* **Forest plot of the meta-analysis between *ACE* DD genotype and total cholesterol levels.**

*Figure S3* **Forest plot of the meta-analysis between *ACE* DD genotype and diastolic blood pressure levels.**

*Figure S4* **Forest plot of the meta-analysis between *ACE* DD genotype and body mass index levels.**

*Figure S5* **Forest plot of the meta-analysis between *ACE* DD genotype and waist circumference levels.**

***Table S1*** Characteristics of the included studies.

| **First author, reference** | **Year** | **Country** | **Race** | **Sex** | **Health status** | **Outcomes** |
| --- | --- | --- | --- | --- | --- | --- |
| Temel SG [S1] | 2019 | Turkey | Other ethnicities | M/F | Patients with CAD | TG/TC/LDL-C/HDL-C/FPG |
| Temel SG [S1] | 2019 | Turkey | Other ethnicities | M/F | Healthy individuals | TG/TC/LDL-C/HDL-C/FPG |
| Tkác I [S2] | 2003 | Slovakia | Caucasian | M/F | Patients witht T2DM | TG/TC/LDL-C/HDL-C/SBP |
| Meyer T [S3] | 2020 | Germany | Caucasian | M/F | Patients with CAD | TG/TC/LDL-C/HDL-C |
| Luptáková L [S4] | 2013 | Slovakia | Caucasian | F | Healthy individuals | TG/TC/LDL-C/HDL-C/FPG/SBP/DBP/BMI |
| Khamlaoui W [S5] | 2020 | Tunisia | Other ethnicities | M/F | Patients with obesity | TG/TC/LDL-C/HDL-C/WC |
| Liu A [S6] | 2019 | China | Asian | M/F | Patients with sudden cardiac arrest and CAD | TC/LDL-C/HDL-C/SBP/DBP/BMI |
| Tsukada K [S7] | 1997 | Japan | Asian | M/F | Patients with CAD and control subjects | TG/TC/HDL-C/DBP/BMI/Genotype count for case-control subjects |
| Hubacek JA [S8] | 2000 | Czech Republic | Caucasian | M | Healthy individuals | TG/TC/LDL-C/HDL-C |
| Akin F [S9] | 2010 | Turkey | Other ethnicities | M/F | Patients with obesity | TG/TC/LDL-C/HDL-C/SBP/DBP/BMI/WC |
| Alsaeid M [S10] | 2004 | Kuwait | Other ethnicities | M/F | Patients witht T1DM | TC/LDL-C/HDL-C/DBP |
| Alsaeid M [S10] | 2004 | Kuwait | Other ethnicities | M/F | Healthy individuals | TC/LDL-C/HDL-C/DBP |
| Araz M [S11] | 2001 | Turkey | Other ethnicities | M/F | Patients witht T2DM | TG/TC/LDL-C/HDL-C/SBP |
| Arnett DK [S12] | 2005 | USA | American | M/F | Patients witht CVD | TG/TC/LDL-C/HDL-C/SBP/DBP |
| Bednarska-Makaruk M [S13] | 2005 | Poland | Caucasian | M | Patients witht alcohol-dependent | TG/TC/LDL-C/HDL-C |
| Bhatti GK [S14] | 2017 | India | Other ethnicities | M/F | Patients with CAD | TG/TC/LDL-C/HDL-C/SBP/DBP/BMI |
| Bhatti GK [S14] | 2017 | India | Other ethnicities | M/F | Healthy individuals | TG/TC/LDL-C/HDL-C/SBP/DBP/BMI |
| Eichner JE [S15] | 2001 | USA | American | M | Patients with CVD | TG/TC/HDL-C |
| Eichner JE [S15] | 2001 | USA | American | F | Patients with CVD | TG/TC/HDL-C |
| El-Kabbany ZA [S16] | 2019 | Egypt | Other ethnicities | M/F | Patients with obesity | TG/TC/LDL-C/HDL-C |
| Felehgari V [S17] | 2011 | Iran | Other ethnicities | M/F | Patients with T2DM | TG/TC/LDL-C/HDL-C |
| Felehgari V [S17] | 2011 | Iran | Other ethnicities | M/F | Patients with T2DM | TG/TC/LDL-C/HDL-C |
| Fossum E [S18] | 2001 | Norway | Other ethnicities | M | Healthy individuals | TG/TC |
| Friedl W [S19] | 1995 | Austria | Caucasian | M/F | Patients with CAD | TG/TC/HDL-C |
| Friedl W [S19] | 1995 | Austria | Caucasian | M/F | Healthy individuals | TG/TC/HDL-C |
| Guneri S [S20] | 2005 | Turkey | Other ethnicities | M/F | Patients with CAD | LDL-C/BMI |
| Ha SK [S21] | 2003 | Korea | Asian | M/F | Patients with T2DM | TG/TC/HDL-C |
| Hadjadj S [S22] | 2008 | France | Caucasian | M/F | Patients with T2DM | TC/LDL-C/HDL-C/SBP/DBP |
| Hamelin BA [S23] | 2011 | Canada | Caucasian | M | Patients with PCAD | TG/TC/LDL-C/HDL-C/FPG/DBP/Genotype count for case-control subjects |
| Huang XH [S24] | 1998 | Finland | Caucasian | M/F | Patients with T2DM | TG/TC/HDL-C/FPG/SBP/DBP |
| Huang XH [S24] | 1998 | Finland | Caucasian | M/F | Healthy individuals | TG/TC/HDL-C/FPG/SBP/DBP |
| Huang XH [S25] | 1999 | Finland | Caucasian | M | Healthy individuals | TG/TC/LDL-C/HDL-C/FPG/SBP/DBP |
| Islam MS [S26] | 2006 | Finland | Caucasian | M/F | Healthy individuals | TG/TC/LDL-C/HDL-C |
| Jacobson AM [S27] | 2010 | USA/Canada | Caucasian | M/F | Patients with T1DM | TG/TC/LDL-C/HDL-C |
| Katsuya T [S28] | 1995 | USA | American | M/F | Patients with T2DM | TG/TC/LDL-C/HDL-C/SBP/DBP/BMI |
| Kim K [S29] | 2009 | Korea | Asian | F | Patients with CVD | TC/LDL-C/HDL-C/SBP/DBP/WC |
| Kim K [S30] | 2009 | Korea | Asian | M/F | Healthy individuals | TG/TC/LDL-C/HDL-C/SBP/DBP/WC |
| Kobayashi K [S31] | 1999 | Japan | Asian | M/F | Patients with T2DM | TG/TC/LDL-C/HDL-C |
| Kogawa K [S32] | 1997 | Japan | Asian | M/F | Patients with T2DM | TG/TC/LDL-C/HDL-C/SBP/DBP/BMI |
| Kogawa K [S32] | 1997 | Japan | Asian | M/F | Healthy individuals | TG/TC/LDL-C/HDL-C/SBP/DBP/BMI |
| Kondo H [S33] | 2015 | Japan | Asian | M/F | Healthy individuals | TC/SBP/DBP |
| Moorthy N [S34] | 2007 | India | Other ethnicities | M/F | Patients with CAD | TC/LDL-C/HDL-C/Genotype count for case-control subjects |
| Maitland-van der Zee AH [S35] | 2008 | USA | American | M/F | Patients with hypertension | TC/LDL-C/HDL-C |
| Maitland-van der Zee AH [S35] | 2008 | USA | American | M/F | Patients with hypertension | TC/LDL-C/HDL-C |
| Makris TK [S36] | 2000 | Greece | Caucasian | M/F | Patients with hypertension | TG/TC/LDL-C/HDL-C/SBP/DBP/BMI |
| Mannami T [S37] | 2001 | Japan | Asian | M | Healthy individuals | TG/TC/HDL-C/FPG/SBP/DBP/BMI |
| Mannami T [S37] | 2001 | Japan | Asian | F | Healthy individuals | TG/TC/HDL-C/FPG/SBP/DBP/BMI |
| Marian AJ [S38] | 2000 | USA | American | M/F | Patients with CAD | TG/TC/LDL-C/HDL-C |
| Marian AJ [S38] | 2000 | USA | American | M/F | Patients with CAD | TG/TC/LDL-C/HDL-C |
| Nagi DK [S39] | 1998 | UK | Other ethnicities | M/F | Patients with T2DM | TC/LDL-C/HDL-C |
| Nagi DK [S39] | 1998 | UK | Other ethnicities | M/F | Healthy individuals | TC/LDL-C/HDL-C |
| Nakai K [S40] | 1994 | Japan | Asian | M/F | Patients with CAD | TC/HDL-C/Genotype count for case-control subjects |
| Nordestgaard BG [S41] | 2010 | Denmark/Finland/Norway/Sweden | Caucasian | M/F | Patients with hypertension | TC |
| Okuno S [S42] | 1992 | Japan | Asian | M/F | Patients with T2DM | TG/TC/HDL-C |
| Okura Y [S43] | 2003 | Japan | Asian | M/F | Patients with CAD | TG/TC/LDL-C/HDL-C |
| Oren I [S44] | 1999 | Israel | Other ethnicities | M | Healthy individuals | TG/TC/LDL-C/HDL-C/SBP/DBP/BMI |
| Passaro A [S45] | 2011 | Italy | Caucasian | M/F | Patients with MetS | TC/LDL-C/HDL-C/SBP/DBP/BMI |
| Pedrinelli R [S46] | 2006 | Italy | Caucasian | M | Patients with hypertension | TG/TC/LDL-C/HDL-C/FPG/SBP/DBP/BMI |
| Prasad A [S47] | 2000 | USA | American | M/F | Patients with coronary atherosclerosis | TC/HDL-C/DBP |
| Sayed-Tabatabaei FA [S48] | 2005 | The Netherlands | Caucasian | M/F | Healthy individuals | TC/HDL-C/SBP/DBP |
| Tseng CH [S49] | 2012 | China | Asian | M/F | Patients with T2DM | TC/LDL-C/HDL-C |
| Uemura K [S50] | 2000 | Japan | Asian | M | Healthy individuals | TG/TC/LDL-C/HDL-C/FPG/SBP/DBP/BMI |
| Mittal G [S51] | 2011 | India | Other ethnicities | M/F | Patients with MetS | WC |
| Herrera CL [S52] | 2016 | Chile | Other ethnicities | M/F | Patients with MetS | WC |
| Herrera CL [S52] | 2016 | Chile | Other ethnicities | M/F | Healthy individuals | WC |
| Das M [S53] | 2008 | India | Other ethnicities | M/F | Healthy individuals | WC |
| Montes-de-Oca-García A [S54] | 2021 | Spain | Caucasian | M/F | Healthy individuals | WC |
| Lemes VA [S55] | 2013 | Brazil | Other ethnicities | M | Patients with obesity | WC |
| Lemes VA [S55] | 2013 | Brazil | Other ethnicities | F | Patients with obesity | WC |
| Jhawat V [S56] | 2019 | India | Other ethnicities | M/F | Patients with hypertension | WC |
| Alvarez R [S57] | 2001 | Spain | Caucasian | M | Patients with PCAD | Genotype count for case-control subjects |
| Alvarez R [S58] | 1998 | Spain | Caucasian | M/F | Patients with PCAD | Genotype count for case-control subjects |
| Bøhn M [S59] | 1993 | Norway | Caucasian | M | Patients with PCAD | Genotype count for case-control subjects |
| Bøhn M [S59] | 1993 | Norway | Caucasian | F | Patients with PCAD | Genotype count for case-control subjects |
| Mohammad AM [S60] | 2020 | Iraq | Other ethnicities | M/F | Patients with PCAD | Genotype count for case-control subjects |
| Yý Lmaz Çiftdoð An D [S61] | 2014 | Turkey | Other ethnicities | M/F | Patients with PCAD | Genotype count for case-control subjects |
| Abd El-Aziz TA [S62] | 2012 | Egypt | Other ethnicities | M/F | Patients with PCAD | Genotype count for case-control subjects |
| Agirbasli M [S63] | 2011 | Turkey | Other ethnicities | M/F | Patients with PCAD | Genotype count for case-control subjects |
| Batalla A [S64] | 2000 | Spain | Caucasian | M | Patients with PCAD | Genotype count for case-control subjects |
| Berdeli A [S65] | 2005 | Turkey | Other ethnicities | M/F | Patients with PCAD | Genotype count for case-control subjects |
| Biggart S [S66] | 1998 | UK | Caucasian | M/F | Patients with PCAD | Genotype count for case-control subjects |
| van Bockxmeer FM [S67] | 2000 | Australia | Caucasian | M/F | Patients with PCAD | Genotype count for case-control subjects |
| Ermis C [S68] | 2002 | Turkey | Other ethnicities | M/F | Patients with PCAD | Genotype count for case-control subjects |
| McCarthy JJ [S69] | 2004 | USA | Caucasian | M/F | Patients with PCAD | Genotype count for case-control subjects |
| Miettinen HE [S70] | 1994 | Finland | Caucasian | M/F | Patients with PCAD | Genotype count for case-control subjects |
| Rallidis LS [S71] | 2009 | Greece | Caucasian | M/F | Patients with PCAD | Genotype count for case-control subjects |
| Ramasawmy R [S72] | 1996 | France | Caucasian | M | Patients with PCAD | Genotype count for case-control subjects |
| Sekuri C [S73] | 2005 | Turkey | Other ethnicities | M/F | Patients with PCAD | Genotype count for case-control subjects |
| Vaisi-Raygani A [S74] | 2010 | Iran | Other ethnicities | M/F | Patients with PCAD | Genotype count for case-control subjects |
| Poorzand H [S75] | 2023 | Iran | Other ethnicities | M/F | Patients with PCAD | Genotype count for case-control subjects |
| Park HY [S76] | 1997 | Korea | Asian | M/F | Patients with CAD | Genotype count for case-control subjects |
| Tran DC [S77] | 2023 | Vietnam | Asian | M/F | Patients with CAD | Genotype count for case-control subjects |
| Ferrari M [S78] | 2002 | Germany/Sweden/Greece/UK | Caucasian | M/F | Patients with CAD | Genotype count for case-control subjects |
| Gardemann A [S79] | 1998 | Germany | Caucasian | M/F | Patients with CAD | Genotype count for case-control subjects |
| Karayannis G [S80] | 2010 | Greece | Caucasian | M/F | Patients with CAD | Genotype count for case-control subjects |
| Koch W [S81] | 2000 | Germany/Sweden/Greece/UK | Caucasian | M/F | Patients with CAD | Genotype count for case-control subjects |
| Nakauchi Y [S82] | 1996 | Japan | Asian | M/F | Patients with CAD | Genotype count for case-control subjects |
| Narne P [S83] | 2012 | India | Other ethnicities | M/F | Patients with CAD | Genotype count for case-control subjects |
| Niemiec P [S84] | 2008 | Poland | Caucasian | M/F | Patients with CAD | Genotype count for case-control subjects |
| Qiu C [S85] | 2007 | China | Asian | M/F | Patients with CAD | Genotype count for case-control subjects |
| Vaisi-Raygani A [S86] | 2012 | Iran | Other ethnicities | M/F | Patients with CAD | Genotype count for case-control subjects |
| Vaisi-Raygani A [S86] | 2012 | Iran | Other ethnicities | M/F | Patients with CAD | Genotype count for case-control subjects |
| Sigusch HH [S87] | 1997 | Germany/Sweden/Greece/UK | Caucasian | M/F | Patients with CAD | Genotype count for case-control subjects |
| Wang XL [S88] | 1996 | Australia | Caucasian | M | Patients with CAD | Genotype count for case-control subjects |
| Wang XL [S88] | 1996 | Australia | Caucasian | F | Patients with CAD | Genotype count for case-control subjects |
| Pfohl M [S89] | 1998 | Germany/Sweden/Greece/UK | Caucasian | M/F | Patients with CAD | Genotype count for case-control subjects |
| Dzimiri N [S90] | 2000 | Saudi Arabia | Other ethnicities | M | Patients with CAD | Genotype count for case-control subjects |
| Pfohl M [S91] | 1999 | Germany | Caucasian | M | Patients with CAD | Genotype count for case-control subjects |
| Pfohl M [S91] | 1999 | Germany | Caucasian | F | Patients with CAD | Genotype count for case-control subjects |

M: male; F: female; PCAD: premature coronary artery disease; CAD: coronary artery disease; T1DM: type 1 diabetes mellitus; T2DM: type 2 diabetes mellitus; CVD: cardiovascular disease; Mets: Metabolic Syndrome; OSAHS: obstructive sleep apnea hypopnea syndrome patients; TG: triglycerides; TC: total cholesterol; LDL-C: low-density lipoprotein cholesterol; HDL-C: high-density lipoprotein cholesterol; FPG: fasting plasma glucose; SBP: systolic blood pressure; DBP: diastolic blood pressure; BMI: body mass index; WC: waist circumference.

***Table S2* Checklist of items to include when reporting a systematic review or meta-analysis**.

| **Section and Topic** | **Item #** | **Checklist item** | **Location where item is reported** |
| --- | --- | --- | --- |
| **TITLE** | | |  |
| Title | 1 | Identify the report as a systematic review. | 1 |
| **ABSTRACT** | | |  |
| Abstract | 2 | See the PRISMA 2020 for Abstracts checklist. | 2 |
| **INTRODUCTION** | | |  |
| Rationale | 3 | Describe the rationale for the review in the context of existing knowledge. | 3-5 |
| Objectives | 4 | Provide an explicit statement of the objective(s) or question(s) the review addresses. | 5 |
| **METHODS** | | |  |
| Eligibility criteria | 5 | Specify the inclusion and exclusion criteria for the review and how studies were grouped for the syntheses. | 13 |
| Information sources | 6 | Specify all databases, registers, websites, organisations, reference lists and other sources searched or consulted to identify studies. Specify the date when each source was last searched or consulted. | 12 |
| Search strategy | 7 | Present the full search strategies for all databases, registers and websites, including any filters and limits used. | 12 |
| Selection process | 8 | Specify the methods used to decide whether a study met the inclusion criteria of the review, including how many reviewers screened each record and each report retrieved, whether they worked independently, and if applicable, details of automation tools used in the process. | 13 |
| Data collection process | 9 | Specify the methods used to collect data from reports, including how many reviewers collected data from each report, whether they worked independently, any processes for obtaining or confirming data from study investigators, and if applicable, details of automation tools used in the process. | 14 |
| Data items | 10a | List and define all outcomes for which data were sought. Specify whether all results that were compatible with each outcome domain in each study were sought (e.g. for all measures, time points, analyses), and if not, the methods used to decide which results to collect. | 14 |
|  | 10b | List and define all other variables for which data were sought (e.g. participant and intervention characteristics, funding sources). Describe any assumptions made about any missing or unclear information. | 14 |
| Study risk of bias assessment | 11 | Specify the methods used to assess risk of bias in the included studies, including details of the tool(s) used, how many reviewers assessed each study and whether they worked independently, and if applicable, details of automation tools used in the process. | 16 |
| Effect measures | 12 | Specify for each outcome the effect measure(s) (e.g. risk ratio, mean difference) used in the synthesis or presentation of results. | 15 |
| Synthesis methods | 13a | Describe the processes used to decide which studies were eligible for each synthesis (e.g. tabulating the study intervention characteristics and comparing against the planned groups for each synthesis (item #5)). | 15 |
|  | 13b | Describe any methods required to prepare the data for presentation or synthesis, such as handling of missing summary statistics, or data conversions. | 15 |
|  | 13c | Describe any methods used to tabulate or visually display results of individual studies and syntheses. | 15 |
|  | 13d | Describe any methods used to synthesize results and provide a rationale for the choice(s). If meta-analysis was performed, describe the model(s), method(s) to identify the presence and extent of statistical heterogeneity, and software package(s) used. | 15 |
|  | 13e | Describe any methods used to explore possible causes of heterogeneity among study results (e.g. subgroup analysis, meta-regression). | 16 |
|  | 13f | Describe any sensitivity analyses conducted to assess robustness of the synthesized results. | 16 |
| Reporting bias assessment | 14 | Describe any methods used to assess risk of bias due to missing results in a synthesis (arising from reporting biases). | 16 |
| Certainty assessment | 15 | Describe any methods used to assess certainty (or confidence) in the body of evidence for an outcome. | 16 |
| **RESULTS** | | |  |
| Study selection | 16a | Describe the results of the search and selection process, from the number of records identified in the search to the number of studies included in the review, ideally using a flow diagram. | 5 |
|  | 16b | Cite studies that might appear to meet the inclusion criteria, but which were excluded, and explain why they were excluded. | 5 |
| Study characteristics | 17 | Cite each included study and present its characteristics. | 5 |
| Risk of bias in studies | 18 | Present assessments of risk of bias for each included study. | 6 |
| Results of individual studies | 19 | For all outcomes, present, for each study: (a) summary statistics for each group (where appropriate) and (b) an effect estimate and its precision (e.g. confidence/credible interval), ideally using structured tables or plots. | 5,6 |
| Results of syntheses | 20a | For each synthesis, briefly summarise the characteristics and risk of bias among contributing studies. | 5,6 |
|  | 20b | Present results of all statistical syntheses conducted. If meta-analysis was done, present for each the summary estimate and its precision (e.g. confidence/credible interval) and measures of statistical heterogeneity. If comparing groups, describe the direction of the effect. | 5,6 |
|  | 20c | Present results of all investigations of possible causes of heterogeneity among study results. | 6 |
|  | 20d | Present results of all sensitivity analyses conducted to assess the robustness of the synthesized results. | 6 |
| Reporting biases | 21 | Present assessments of risk of bias due to missing results (arising from reporting biases) for each synthesis assessed. | 6 |
| Certainty of evidence | 22 | Present assessments of certainty (or confidence) in the body of evidence for each outcome assessed. | 6 |
| **DISCUSSION** | | |  |
| Discussion | 23a | Provide a general interpretation of the results in the context of other evidence. | 7 |
|  | 23b | Discuss any limitations of the evidence included in the review. | 11 |
|  | 23c | Discuss any limitations of the review processes used. | 11 |
|  | 23d | Discuss implications of the results for practice, policy, and future research. | 11 |
| **OTHER INFORMATION** | | |  |
| Registration and protocol | 24a | Provide registration information for the review, including register name and registration number, or state that the review was not registered. | - |
|  | 24b | Indicate where the review protocol can be accessed, or state that a protocol was not prepared. | - |
|  | 24c | Describe and explain any amendments to information provided at registration or in the protocol. | - |
| Support | 25 | Describe sources of financial or non-financial support for the review, and the role of the funders or sponsors in the review. | 23 |
| Competing interests | 26 | Declare any competing interests of review authors. | 23 |
| Availability of data, code and other materials | 27 | Report which of the following are publicly available and where they can be found: template data collection forms; data extracted from included studies; data used for all analyses; analytic code; any other materials used in the review. | 23 |


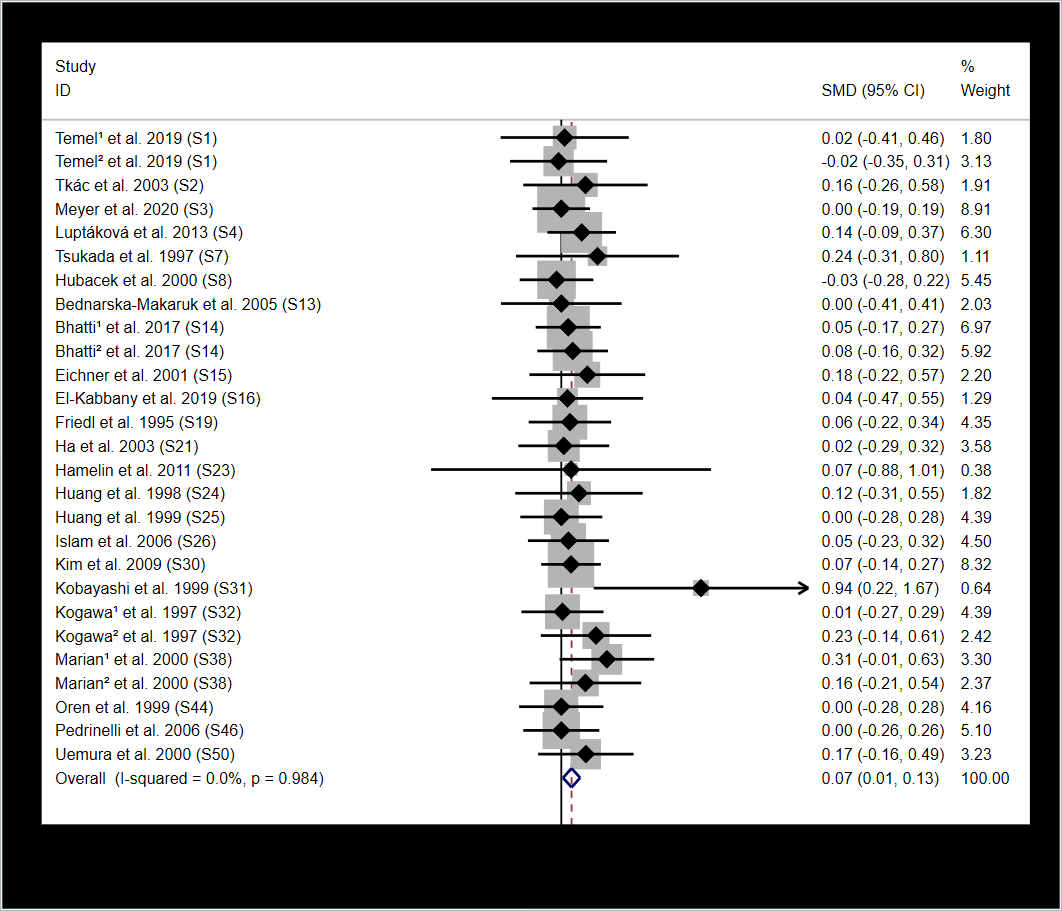


*Figure S1* **Forest plot of the meta-analysis between *ACE* DD genotype and triglycerides levels.**

*
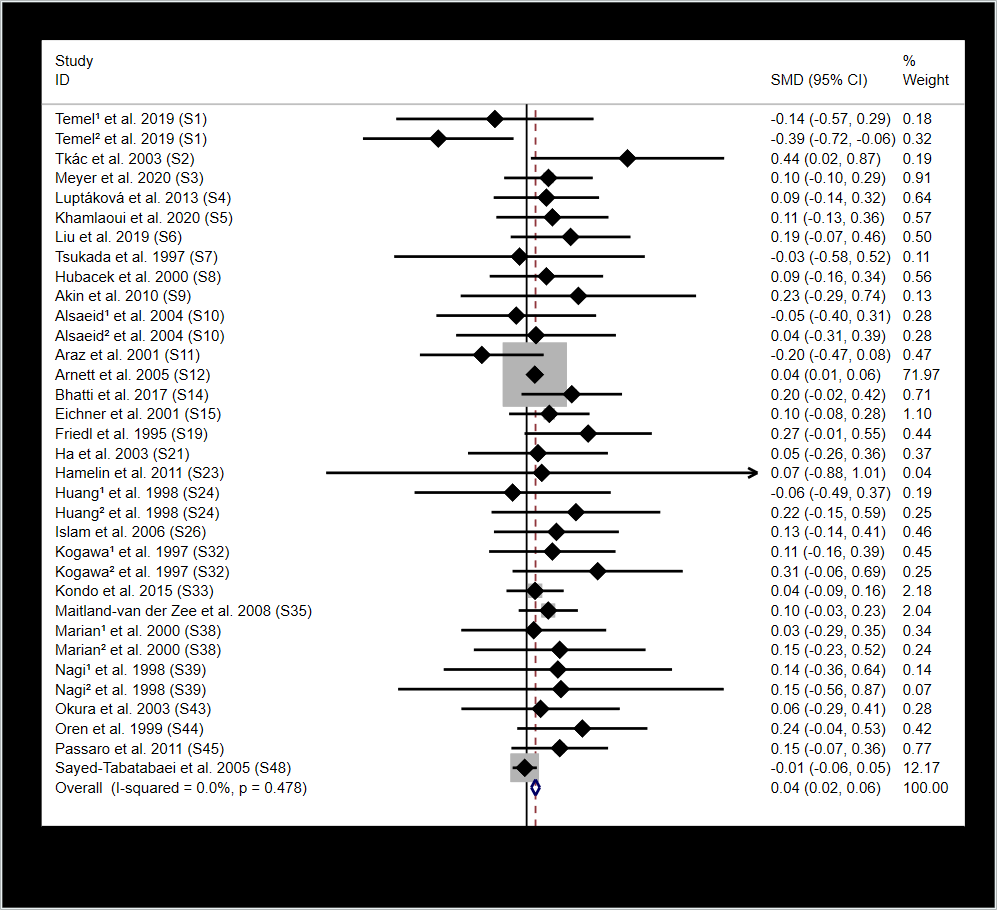
*

*Figure S2* **Forest plot of the meta-analysis between *ACE* DD genotype and total cholesterol levels.**

**
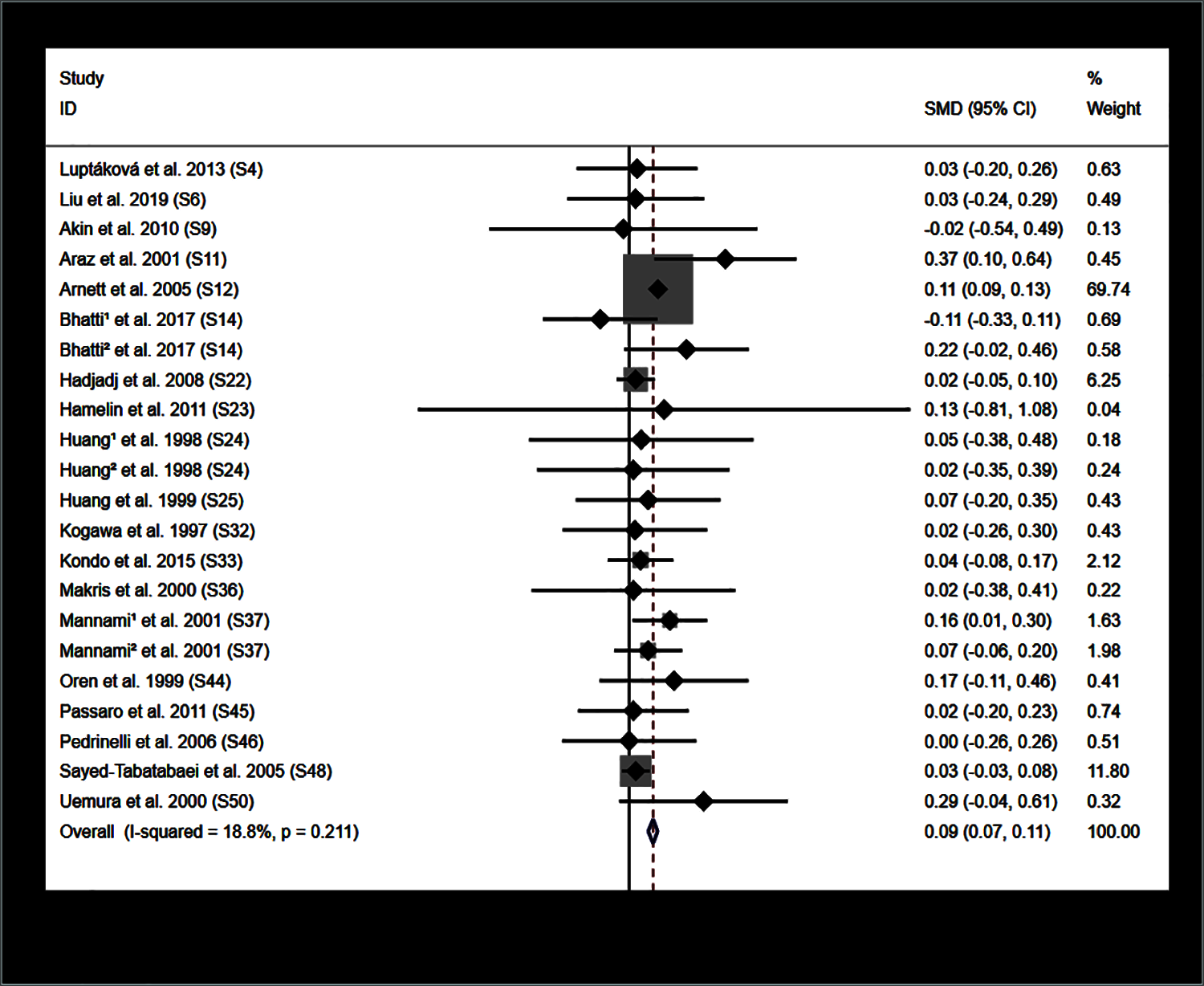
**

*Figure S3* **Forest plot of the meta-analysis between *ACE* DD genotype and diastolic blood pressure levels.**

**
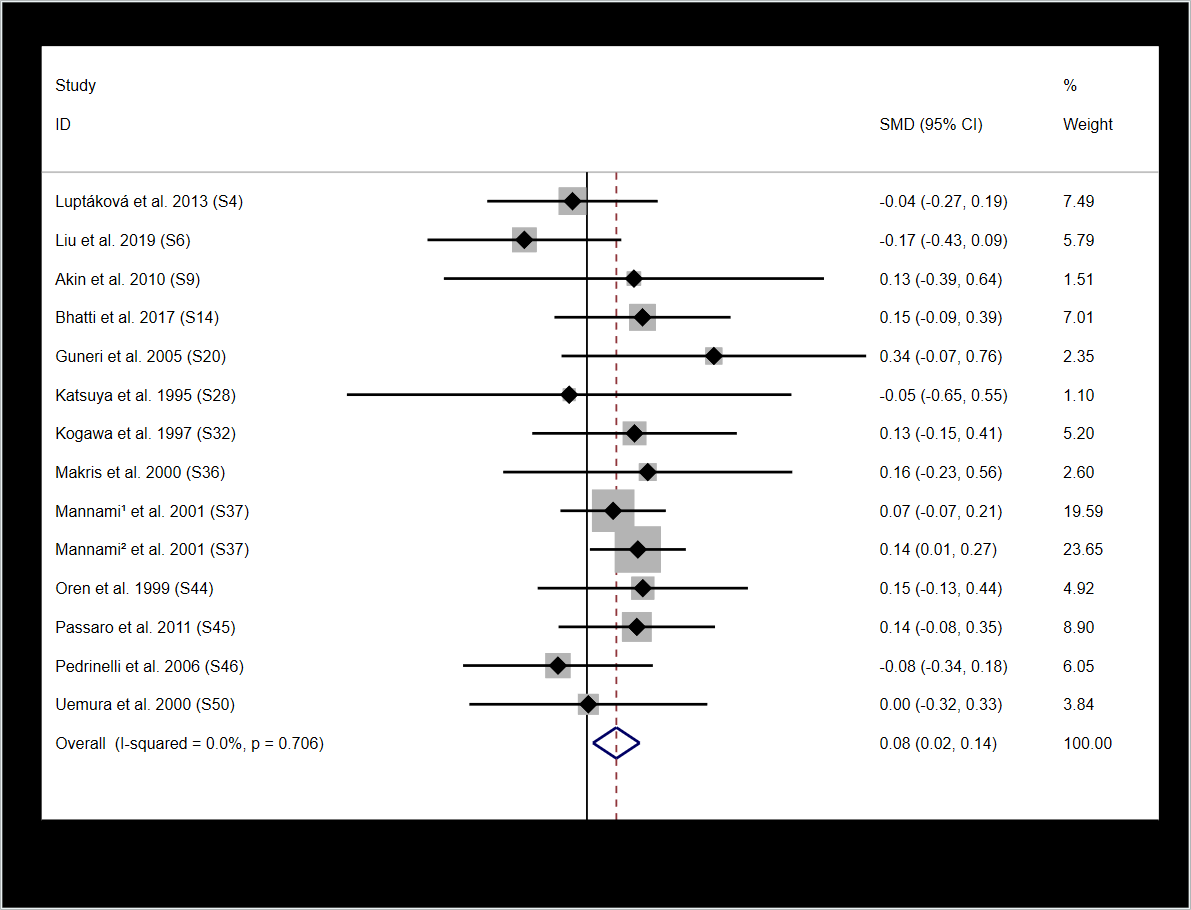
**

*Figure S4* **Forest plot of the meta-analysis between *ACE* DD genotype and body mass index levels.**

**
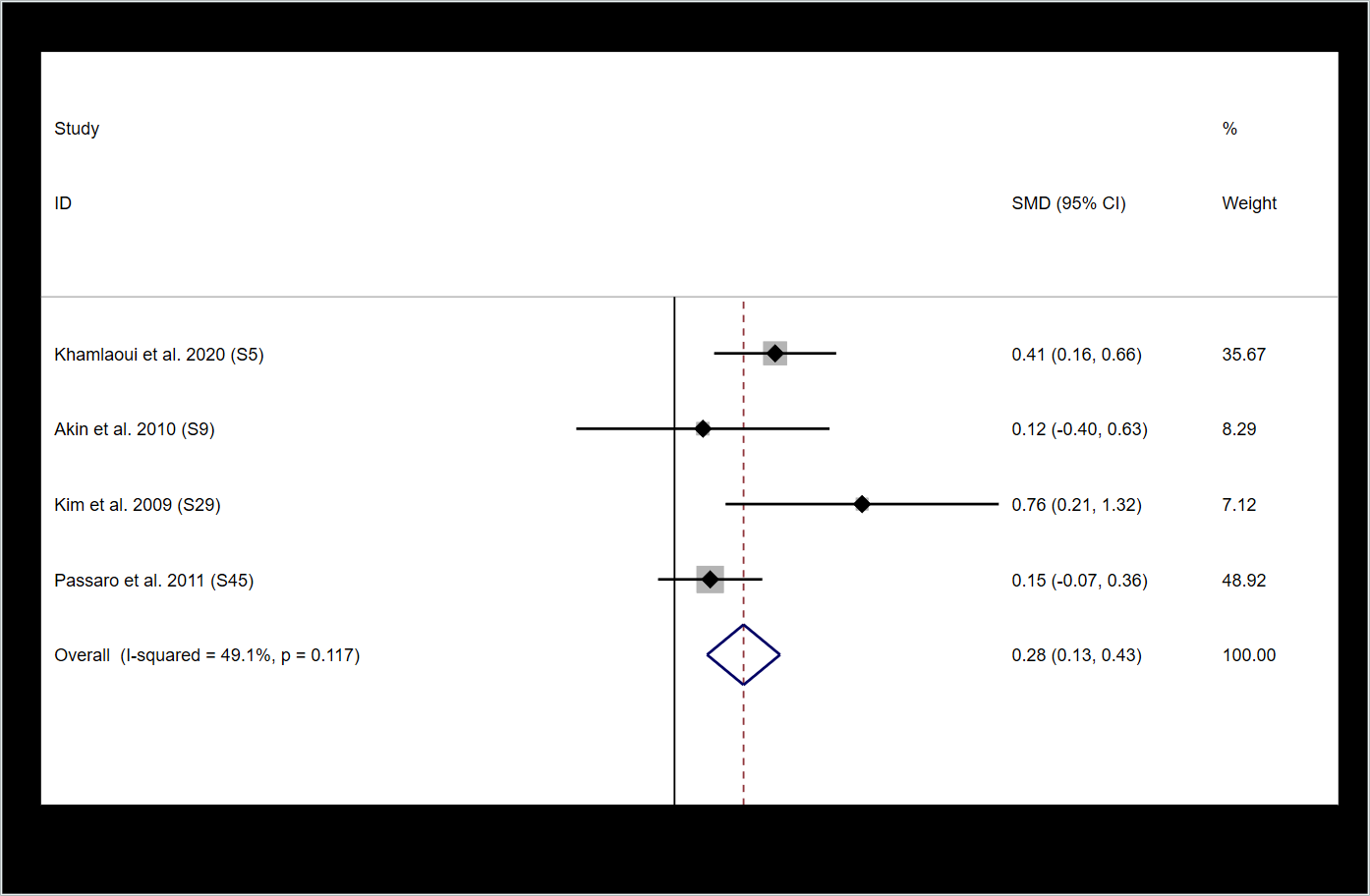
**

*Figure S5* **Forest plot of the meta-analysis between *ACE* DD genotype and waist circumference levels.**

**References**

S1. Temel SG, Ergoren MC, Yilmaz I, Oral HB. The use of ACE INDEL polymorphism as a biomarker of coronary artery disease (CAD) in humans with Mediterranean-style diet. Int J Biol Macromol. 2019;123:576-580. doi: 10.1016/j.ijbiomac.2018.11.021.

S2. Tkác I, Salagovic J, Kozárová M, Rosolová H, Molcányiová A, Mosorjáková D, Chleborádová M, Kalina I. Interaction between angiotensin-converting enzyme genotype and glycaemic control influences lipoprotein levels in type 2 diabetes mellitus. Wien Klin Wochenschr. 2003;115(1-2):36-40. doi: 10.1007/BF03040270.

S3. Meyer T, Rothe I, Staab J, Deter HC, Fangauf SV, Hamacher S, Hellmich M, Jünger J, Ladwig KH, Michal M, Petrowski K, Ronel J, Söllner W, Weber C, de Zwaan M, Williams RB, Albus C, Herrmann-Lingen C; SPIRR-CAD Investigators. Length Polymorphisms in the Angiotensin I-Converting Enzyme Gene and the Serotonin-Transporter-Linked Polymorphic Region Constitute a Risk Haplotype for Depression in Patients with Coronary Artery Disease. Biochem Genet. 2020;58(4):631-648. doi: 10.1007/s10528-020-09967-w.

S4. Luptáková L, Benčová D, Siváková D, Cvíčelová M. Association of CILP2 and ACE gene polymorphisms with cardiovascular risk factors in Slovak midlife women. Biomed Res Int. 2013;2013:634207. doi: 10.1155/2013/634207.

S5. Khamlaoui W, Mehri S, Hammami S, Elosua R, Hammami M. Association of angiotensin-converting enzyme insertion/deletion (ACE I/D) and angiotensinogen (AGT M235T) polymorphisms with the risk of obesity in a Tunisian population. J Renin Angiotensin Aldosterone Syst. 2020;21(2):1470320320907820. doi: 10.1177/1470320320907820.

S6. Liu A, Wang S, Zhang C, Sun D, Song X. Role of angiotensin-converting enzyme insertion/deletion polymorphism in sudden cardiac arrest. J Cell Biochem. 2019;120(3):3474-3478. doi: 10.1002/jcb.27622.

S7. Tsukada K, Ishimitsu T, Tsuchiya N, Horinaka S, Matsuoka H. Angiotensin-converting enzyme gene polymorphism and cardiovascular endocrine system in coronary angiography patients. Jpn Heart J. 1997;38(6):799-810. doi: 10.1536/ihj.38.799.

S8. Hubacek JA, Pitha J, Podrapská I, Sochman J, Adámková V, Lánská V, Poledne R. Insertion/deletion polymorphism in the angiotensin-converting enzyme gene in myocardial infarction survivors. Med Sci Monit. 2000;6(3):503-6.

S9. Akin F, Turgut S, Bastemir M, Turgut G, Kursunluoglu R, Karasu U, Guclu A. Angiotensin-converting enzyme gene polymorphism in overweight and obese Turkish patients with insulin resistance. DNA Cell Biol. 2010;29(4):207-12. doi: 10.1089/dna.2009.0934.

S10. Alsaeid M, Moussa MA, Haider MZ, Refai TM, Abdella N, Al-Sheikh N, Gomez JE. Angiotensin-converting enzyme gene polymorphism and lipid profiles in Kuwaiti children with type 1 diabetes. Pediatr Diabetes. 2004;5(2):87-94. doi: 10.1111/j.1399-543X.2004.00040.x.

S11. Araz M, Yilmaz N, Güngör K, Okan V, Kepekci Y, Sükrü Aynacioglu A. Angiotensin-converting enzyme gene polymorphism and microvascular complications in Turkish type 2 diabetic patients. Diabetes Res Clin Pract. 2001;54(2):95-104. doi: 10.1016/s0168-8227(01)00257-1.

S12. Arnett DK, Davis BR, Ford CE, Boerwinkle E, Leiendecker-Foster C, Miller MB, Black H, Eckfeldt JH. Pharmacogenetic association of the angiotensin-converting enzyme insertion/deletion polymorphism on blood pressure and cardiovascular risk in relation to antihypertensive treatment: the Genetics of Hypertension-Associated Treatment (GenHAT) study. Circulation. 2005;111(25):3374-83. doi: 10.1161/CIRCULATIONAHA.104.504639.

S13. Bednarska-Makaruk M, Rodo M, Markuszewski C, Rozenfeld A, Swiderska M, Habrat B, Wehr H. Polymorphisms of apolipoprotein E and angiotensin-converting enzyme genes and carotid atherosclerosis in heavy drinkers. Alcohol Alcohol. 2005;40(4):274-82. doi: 10.1093/alcalc/agh157.

S14. Bhatti GK, Bhatti JS, Vijayvergiya R, Singh B. Implications of ACE (I/D) Gene Variants to the Genetic Susceptibility of Coronary Artery Disease in Asian Indians. Indian J Clin Biochem. 2017;32(2):163-170. doi: 10.1007/s12291-016-0588-3.

S15. Eichner JE, Christiansen VJ, Moore WE, Dunn ST, Schechter E. Angiotensin-converting enzyme gene polymorphism in a cohort of coronary angiography patients. Atherosclerosis. 2001;154(3):673-9. doi: 10.1016/s0021-9150(00)00395-6.

S16. El-Kabbany ZA, Hamza RT, Shinkar DM, Kamal TM, Abdelmageed RI, Said MS, Abdel-Hamid MI. Screening of Egyptian obese children and adolescents for insertion/deletion (I/D) polymorphism in angiotensin-converting enzyme gene. Int J Pediatr Adolesc Med. 2019;6(1):21-24. doi: 10.1016/j.ijpam.2019.02.008.

S17. Felehgari V, Rahimi Z, Mozafari H, Vaisi-Raygani A. ACE gene polymorphism and serum ACE activity in Iranians type II diabetic patients with macroalbuminuria. Mol Cell Biochem. 2011;346(1-2):23-30. doi: 10.1007/s11010-010-0587-2.

S18. Fossum E, Berge KE, Høieggen A, Moan A, Rostrup M, Kjeldsen SE, Eide I, Berg K. Polymorphisms in candidate genes for blood pressure regulation in young men with normal or elevated screening blood pressure. Blood Press. 2001;10(2):92-100. doi: 10.1080/08037050152112078.

S19. Friedl W, Krempler F, Paulweber B, Pichler M, Sandhofer F. A deletion polymorphism in the angiotensin converting enzyme gene is not associated with coronary heart disease in an Austrian population. Atherosclerosis. 1995;112(2):137-43. doi: 10.1016/0021-9150(94)05406-9.

S20. Guneri S, Baris N, Aytekin D, Akdeniz B, Pekel N, Bozdemir V. The relationship between angiotensin converting enzyme gene polymorphism, coronary artery disease, and stent restenosis: the role of angiotensin converting enzyme inhibitors in stent restenosis in patients with diabetes mellitus. Int Heart J. 2005;46(5):889-97. doi: 10.1536/ihj.46.889.

S21. Ha SK, Park HC, Park HS, Kang BS, Lee TH, Hwang HJ, Kim SJ, Kim DH, Kang SW, Choi KH, Lee HY, Han DS. ACE gene polymorphism and progression of diabetic nephropathy in Korean type 2 diabetic patients: effect of ACE gene DD on the progression of diabetic nephropathy. Am J Kidney Dis. 2003;41(5):943-9. doi: 10.1016/s0272-6386(03)00191-4.

S22. Hadjadj S, Fumeron F, Roussel R, Saulnier PJ, Gallois Y, Ankotche A, Travert F, Abi Khalil C, Miot A, Alhenc-Gelas F, Lievre M, Marre M; DIABHYCAR Study Group; DIAB2NEPHROGENE Study Group; SURDIAGENE Study Group. Prognostic value of the insertion/deletion polymorphism of the ACE gene in type 2 diabetic subjects: results from the Non-insulin-dependent Diabetes, Hypertension, Microalbuminuria or Proteinuria, Cardiovascular Events, and Ramipril (DIABHYCAR), Diabete de type 2, Nephropathie et Genetique (DIAB2NEPHROGENE), and Survie, Diabete de type 2 et Genetique (SURDIAGENE) studies. Diabetes Care. 2008;31(9):1847-52. doi: 10.2337/dc07-2079.

S23. Hamelin BA, Zakrzewski-Jakubiak M, Robitaille NM, Bogaty P, Labbé L, Turgeon J. Increased risk of myocardial infarction associated with angiotensin-converting enzyme gene polymorphism is age dependent. J Clin Pharmacol. 2011;51(9):1286-92. doi: 10.1177/0091270010382420.

S24. Huang XH, Rantalaiho V, Wirta O, Pasternack A, Koivula T, Hiltunen T, Nikkari T, Lehtimäki T. Relationship of the angiotensin-converting enzyme gene polymorphism to glucose intolerance, insulin resistance, and hypertension in NIDDM. Hum Genet. 1998;102(3):372-8. doi: 10.1007/s004390050707.

S25. Huang XH, Loimaala A, Nenonen A, Mercuri M, Vuori I, Pasanen M, Oja P, Bond G, Koivula T, Hiltunen TP, Nikkari T, Lehtimäki T. Relationship of angiotensin-converting enzyme gene polymorphism to carotid wall thickness in middle-aged men. J Mol Med (Berl). 1999;77(12):853-8. doi: 10.1007/s001099900061.

S26. Islam MS, Lehtimäki T, Juonala M, Kähönen M, Hutri-Kähönen N, Kainulainen K, Miettinen H, Taittonen L, Kontula K, Viikari JS, Raitakari OT. Polymorphism of the angiotensin-converting enzyme (ACE) and angiotesinogen (AGT) genes and their associations with blood pressure and carotid artery intima media thickness among healthy Finnish young adults--the Cardiovascular Risk in Young Finns Study. Atherosclerosis. 2006;188(2):316-22. doi: 10.1016/j.atherosclerosis.2005.11.008.

S27. Jacobson AM, Paterson AD, Ryan CM, Cleary PA, Waberski BH, Weinger K, Musen G, Dahms W, Bayless M, Silvers N, Harth J, Boright AP; DCCT/EDIC Research Group. The associations of apolipoprotein E and angiotensin-converting enzyme polymorphisms and cognitive function in Type 1 diabetes based on an 18-year follow-up of the DCCT cohort. Diabet Med. 2010;27(1):15-22. doi: 10.1111/j.1464-5491.2009.02885.x.

S28. Katsuya T, Horiuchi M, Chen YD, Koike G, Pratt RE, Dzau VJ, Reaven GM. Relations between deletion polymorphism of the angiotensin-converting enzyme gene and insulin resistance, glucose intolerance, hyperinsulinemia, and dyslipidemia. Arterioscler Thromb Vasc Biol. 1995;15(6):779-82. doi: 10.1161/01.atv.15.6.779.

S29. Kim K. Association of angiotensin-converting enzyme insertion/deletion polymorphism with obesity, cardiovascular risk factors and exercise-mediated changes in Korean women. Eur J Appl Physiol. 2009;105(6):879-87. doi: 10.1007/s00421-008-0973-6.

S30. Kim K, Ahn N, Park J, Koh J, Jung S, Kim S, Moon S. Association of angiotensin-converting enzyme I/D and α-actinin-3 R577X genotypes with metabolic syndrome risk factors in Korean children. Obes Res Clin Pract. 2016;10 Suppl 1:S125-S132. doi: 10.1016/j.orcp.2015.09.008.

S31. Kobayashi K, Amemiya S, Mochizuki M, Kobayashi K, Matsushita K, Sawanobori E, Ishihara T, Higashida K, Shimura Y, Kato K, Nakazawa S. Association of angiotensin-converting enzyme gene polymorphism with lipid profiles in children and adolescents with insulin-dependent diabetes mellitus. Horm Res. 1999;51(4):201-4. doi: 10.1159/000023358.

S32. Kogawa K, Nishizawa Y, Hosoi M, Kawagishi T, Maekawa K, Shoji T, Okuno Y, Morii H. Effect of polymorphism of apolipoprotein E and angiotensin-converting enzyme genes on arterial wall thickness. Diabetes. 1997;46(4):682-7. doi: 10.2337/diab.46.4.682.

S33. Kondo H, Ninomiya T, Hata J, Hirakawa Y, Yonemoto K, Arima H, Nagata M, Tsuruya K, Kitazono T, Kiyohara Y. Angiotensin I-converting enzyme gene polymorphism enhances the effect of hypercholesterolemia on the risk of coronary heart disease in a general Japanese population: the hisayama study. J Atheroscler Thromb. 2015;22(4):390-403. doi: 10.5551/jat.24166.

S34. Moorthy N, Saligrama Ramegowda K, Jain S, Bharath G, Sinha A, Nanjappa MC, Christopher R. Role of Angiotensin-Converting Enzyme (ACE) gene polymorphism and ACE activity in predicting outcome after acute myocardial infarction. Int J Cardiol Heart Vasc. 2021;32:100701. doi: 10.1016/j.ijcha.2020.100701. eCollection 2021 Feb.

S35. Maitland-van der Zee AH, Boerwinkle E, Arnett DK, Davis BR, Leiendecker-Foster C, Miller MB, Klungel OH, Ford CE, Eckfeldt JH. Absence of an interaction between the angiotensin-converting enzyme insertion-deletion polymorphism and pravastatin on cardiovascular disease in high-risk hypertensive patients: the Genetics of Hypertension-Associated Treatment (GenHAT) study. Am Heart J. 2007;153(1):54-8. doi: 10.1016/j.ahj.2006.10.019.

S36. Makris TK, Stavroulakis GA, Dafni UG, Gialeraki AE, Krespi PG, Hatzizacharias AN, Tsoukala CG, Vythoulkas JS, Kyriakidis MK. ACE/DD genotype is associated with hemostasis balance disturbances reflecting hypercoagulability and endothelial dysfunction in patients with untreated hypertension. Am Heart J. 2000;140(5):760-5. doi: 10.1067/mhj.2000.110764.

S37. Mannami T, Katsuya T, Baba S, Inamoto N, Ishikawa K, Higaki J, Ogihara T, Ogata J. Low potentiality of angiotensin-converting enzyme gene insertion/deletion polymorphism as a useful predictive marker for carotid atherogenesis in a large general population of a Japanese city: the Suita study. Stroke. 2001;32(6):1250-6. doi: 10.1161/01.str.32.6.1250.

S38. Marian AJ, Safavi F, Ferlic L, Dunn JK, Gotto AM, Ballantyne CM. Interactions between angiotensin-I converting enzyme insertion/deletion polymorphism and response of plasma lipids and coronary atherosclerosis to treatment with fluvastatin: the lipoprotein and coronary atherosclerosis study. J Am Coll Cardiol. 2000;35(1):89-95. doi: 10.1016/s0735-1097(99)00535-5.

S39. Nagi DK, Foy CA, Mohamed-Ali V, Yudkin JS, Grant PJ, Knowler WC. Angiotensin-1-converting enzyme (ACE) gene polymorphism, plasma ACE levels, and their association with the metabolic syndrome and electrocardiographic coronary artery disease in Pima Indians. Metabolism. 1998;47(5):622-6. doi: 10.1016/s0026-0495(98)90250-5.

S40. Nakai K, Itoh C, Miura Y, Hotta K, Musha T, Itoh T, Miyakawa T, Iwasaki R, Hiramori K. Deletion polymorphism of the angiotensin I-converting enzyme gene is associated with serum ACE concentration and increased risk for CAD in the Japanese. Circulation. 1994;90(5):2199-202. doi: 10.1161/01.cir.90.5.2199.

S41. Nordestgaard BG, Kontula K, Benn M, Dahlöf B, de Faire U, Edelman JM, Eliasson E, Fyhrquist F, Hille DA, Ibsen H, Lyle PA, Berg K, Sandberg M, Sethi AA, Wong PH, Os I. Effect of ACE insertion/deletion and 12 other polymorphisms on clinical outcomes and response to treatment in the LIFE study. Pharmacogenet Genomics. 2010;20(2):77-85. doi: 10.1097/FPC.0b013e328333f70b.

S42. Okuno S, Utsugi T, Ohno T, Ohyama Y, Uchiyama T, Tomono S, Kurabayashi M. Angiotensin-converting enzyme gene polymorphism as a potent risk factor for developing microalbuminuria in Japanese patients with type 2 diabetes mellitus: a 9-year follow-up study. J Int Med Res. 2003;31(4):290-8. doi: 10.1177/147323000303100406.

S43. Okura Y, Hayashi K, Shingu T, Kuga Y, Nomura S, Kajiyama G, Nakashima Y, Saku K. Angiotensin-converting enzyme insertion/deletion genotype is associated with the activities of plasma coagulation factor VII and X independent of triglyceride metabolism. Coron Artery Dis. 2003;14(4):285-91. doi: 10.1097/01.mca.0000072847.84236.34.

S44. Oren I, Brook JG, Gershoni-Baruch R, Kepten I, Tamir A, Linn S, Wolfovitz E. The D allele of the angiotensin-converting enzyme gene contributes towards blood LDL-cholesterol levels and the presence of hypertension. Atherosclerosis. 1999;145(2):267-71. doi: 10.1016/s0021-9150(99)00075-1.

S45. Passaro A, Dalla Nora E, Marcello C, Di Vece F, Morieri ML, Sanz JM, Bosi C, Fellin R, Zuliani G. PPARγ Pro12Ala and ACE ID polymorphisms are associated with BMI and fat distribution, but not metabolic syndrome. Cardiovasc Diabetol. 2011;10:112. doi: 10.1186/1475-2840-10-112.

S46. Pedrinelli R, Dell'Omo G, Penno G, Di Bello V, Pucci L, Fotino C, Lucchesi D, Del Prato S, Dal Fiume C, Barlassina C, Cusi D. Alpha-adducin and angiotensin-converting enzyme polymorphisms in hypertension: evidence for a joint influence on albuminuria. J Hypertens. 2006;24(5):931-7. doi: 10.1097/01.hjh.0000222764.92229.6d.

S47. Prasad A, Narayanan S, Husain S, Padder F, Waclawiw M, Epstein N, Quyyumi AA. Insertion-deletion polymorphism of the ACE gene modulates reversibility of endothelial dysfunction with ACE inhibition. Circulation. 2000;102(1):35-41. doi: 10.1161/01.cir.102.1.35.

S48. Sayed-Tabatabaei FA, Schut AF, Arias Vásquez A, Bertoli-Avella AM, Hofman A, Witteman JC, van Duijn CM. Angiotensin converting enzyme gene polymorphism and cardiovascular morbidity and mortality: the Rotterdam Study. J Med Genet. 2005;42(1):26-30. doi: 10.1136/jmg.2004.022756.

S49. Tseng CH, Tseng FH, Chong CK, Tseng CP, Cheng JC. Angiotensin-converting enzyme genotype and peripheral arterial disease in diabetic patients. Exp Diabetes Res. 2012;2012:698695. doi: 10.1155/2012/698695.

S50. Uemura K, Nakura J, Kohara K, Miki T. Association of ACE I/D polymorphism with cardiovascular risk factors. Hum Genet. 2000;107(3):239-42. doi: 10.1007/s004390000358.

S51. Mittal G, Gupta V, Haque SF, Khan AS. Effect of angiotensin converting enzyme gene I/D polymorphism in patients with metabolic syndrome in North Indian population. Chin Med J (Engl). 2011;124(1):45-8.

S52. Herrera CL, Castillo W, Estrada P, Mancilla B, Reyes G, Saavedra N, Guzmán N, Serón P, Lanas F, Salazar LA. Association of polymorphisms within the Renin-Angiotensin System with metabolic syndrome in a cohort of Chilean subjects. Arch Endocrinol Metab. 2016;60(3):190-8. doi: 10.1590/2359-3997000000134.

S53. Das M, Pal S, Ghosh A. Synergistic effects of ACE (I/D) and Apo E (Hha I) gene polymorphisms on obesity, fat mass, and blood glucose level among the adult Asian Indians: A population-based study from Calcutta, India. Indian J Endocrinol Metab. 2013;17(1):101-4. doi: 10.4103/2230-8210.107816.

S54. Montes-de-Oca-García A, Perez-Bey A, Velázquez-Díaz D, Corral-Pérez J, Opazo-Díaz E, Rebollo-Ramos M, Gómez-Gallego F, Cuenca-García M, Casals C, Ponce-González JG. Influence of ACE Gene I/D Polymorphism on Cardiometabolic Risk, Maximal Fat Oxidation, Cardiorespiratory Fitness, Diet and Physical Activity in Young Adults. Int J Environ Res Public Health. 2021;18(7):3443. doi: 10.3390/ijerph18073443.

S55. Lemes VA, Neves AL, Guazzelli IC, Frazzatto E, Nicolau C, Corrêa-Giannella ML, Velho G, Villares SM. Angiotensin converting enzyme insertion/deletion polymorphism is associated with increased adiposity and blood pressure in obese children and adolescents. Gene. 2013;532(2):197-202. doi: 10.1016/j.gene.2013.09.065.

S56. Jhawat V, Gupta S, Agarwal BK, Roy P, Saini V. Angiotensin Converting Enzyme Gene Insertion/Deletion Polymorphism Is Not Responsible for Antihypertensive Therapy Induced New Onset of Type 2 Diabetes in Essential Hypertension. Clin Med Insights Endocrinol Diabetes. 2019;12:1179551418825037. doi: 10.1177/1179551418825037.

S57. Alvarez R, González P, Batalla A, Reguero JR, Iglesias-Cubero G, Hevia S, Cortina A, Merino E, González I, Alvarez V, Coto E. Association between the NOS3 (-786 T/C) and the ACE (I/D) DNA genotypes and early coronary artery disease. Nitric Oxide. 2001;5(4):343-8. doi: 10.1006/niox.2001.0351.

S58. Alvarez R, Reguero JR, Batalla A, Iglesias-Cubero G, Cortina A, Alvarez V, Coto E. Angiotensin-converting enzyme and angiotensin II receptor 1 polymorphisms: association with early coronary disease. Cardiovasc Res. 1998;40(2):375-9. doi: 10.1016/s0008-6363(98)00179-5.

S59. Bøhn M, Berge KE, Bakken A, Erikssen J, Berg K. Insertion/deletion (I/D) polymorphism at the locus for angiotensin I-converting enzyme and parental history of myocardial infarction. Clin Genet. 1993;44(6):298-301. doi: 10.1111/j.1399-0004.1993.tb03904.x.

S60. Mohammad AM, Othman GO, Saeed CH, Al Allawi S, Gedeon GS, Qadir SM, Al-Allawi N. Genetic polymorphisms in early-onset myocardial infarction in a sample of Iraqi patients: a pilot study. BMC Res Notes. 2020;13(1):541. doi: 10.1186/s13104-020-05367-w.

S61. Yý Lmaz Çiftdoð An D. ACE I/D Gene Polymorphism in Children with Family History of Premature Coronary Disease. Arq Bras Cardiol. 2014;103(5):440-442. doi: 10.5935/abc.20140182.

S62. Abd El-Aziz TA, Hussein YM, Mohamed RH, Shalaby SM. Renin-angiotensin system genes polymorphism in Egyptians with premature coronary artery disease. Gene. 2012;498(2):270-5. doi: 10.1016/j.gene.2012.02.033.

S63. Agirbasli M, Guney AI, Ozturhan HS, Agirbasli D, Ulucan K, Sevinc D, Kirac D, Ryckman KK, Williams SM. Multifactor dimensionality reduction analysis of MTHFR, PAI-1, ACE, PON1, and eNOS gene polymorphisms in patients with early onset coronary artery disease. Eur J Cardiovasc Prev Rehabil. 2011;18(6):803-9. doi: 10.1177/1741826711398806.

S64. Batalla A, Alvarez R, Reguero JR, Hevia S, Iglesias-Cubero G, Alvarez V, Cortina A, González P, Celada MM, Medina A, Coto E. Synergistic effect between apolipoprotein E and angiotensinogen gene polymorphisms in the risk for early myocardial infarction. Clin Chem. 2000;46(12):1910-5.

S65. Berdeli A, Sekuri C, Sirri Cam F, Ercan E, Sagcan A, Tengiz I, Eser E, Akin M. Association between the eNOS (Glu298Asp) and the RAS genes polymorphisms and premature coronary artery disease in a Turkish population. Clin Chim Acta. 2005;351(1-2):87-94. doi: 10.1016/j.cccn.2004.08.015.

S66. Biggart S, Chin D, Fauchon M, Cardew G, du Fou L, Harker N, Quinn E, Keller C, Vincent R, Mayne L. Association of genetic polymorphisms in the ACE, ApoE, and TGF beta genes with early onset ischemic heart disease. Clin Cardiol. 1998;21(11):831-6. doi: 10.1002/clc.4960211109.

S67. van Bockxmeer FM, Mamotte CD, Burke V, Taylor RR. Angiotensin-converting enzyme gene polymorphism and premature coronary heart disease. Clin Sci (Lond). 2000;99(3):247-51. doi: 10.1042/cs20000018.

S68. Ermis C, Tsai MY, Hanson NQ, Akar N, Aras O. Angiotensin I converting enzyme, angiotensin II type 1 receptor and angiotensinogen polymorphisms and early myocardial infarction in Turkish population. Thromb Haemost. 2002;88(4):693-4.

S69. McCarthy JJ, Parker A, Salem R, Moliterno DJ, Wang Q, Plow EF, Rao S, Shen G, Rogers WJ, Newby LK, Cannata R, Glatt K, Topol EJ; GeneQuest Investigators. Large scale association analysis for identification of genes underlying premature coronary heart disease: cumulative perspective from analysis of 111 candidate genes. J Med Genet. 2004;41(5):334-41. doi: 10.1136/jmg.2003.016584.

S70. Miettinen HE, Korpela K, Hämäläinen L, Kontula K. Polymorphisms of the apolipoprotein and angiotensin converting enzyme genes in young North Karelian patients with coronary heart disease. Hum Genet. 1994;94(2):189-92. doi: 10.1007/BF00202868.

S71. Rallidis LS, Gialeraki A, Varounis C, Dagres N, Kotakos C, Travlou A, Lekakis J, Kremastinos DT. Lack of association of angiotensin-converting enzyme insertion/deletion polymorphism and myocardial infarction at very young ages. Biomarkers. 2009;14(6):401-5. doi: 10.1080/13547500903039966.

S72. Ramasawmy R, Manraj M, Kotea N, Shun NK, Genin E, Feingold J, Krishnamoorthy R, Baligadoo S. Lack of association of angiotensin I-converting enzyme gene polymorphism and premature myocardial infarction in Mauritian Indians. Clin Genet. 1996;50(6):551-4. doi: 10.1111/j.1399-0004.1996.tb02737.x.

S73. Sekuri C, Cam FS, Ercan E, Tengiz I, Sagcan A, Eser E, Berdeli A, Akin M. Renin-angiotensin system gene polymorphisms and premature coronary heart disease. J Renin Angiotensin Aldosterone Syst. 2005;6(1):38-42. doi: 10.3317/jraas.2005.005.

S74. Vaisi-Raygani A, Ghaneialvar H, Rahimi Z, Nomani H, Saidi M, Bahrehmand F, Vaisi-Raygani A, Tavilani H, Pourmotabbed T. The angiotensin converting enzyme D allele is an independent risk factor for early onset coronary artery disease. Clin Biochem. 2010;43(15):1189-94. doi: 10.1016/j.clinbiochem.2010.07.010.

S75. Poorzand H, Fazeli B, Khajavi O, Gholoobi A, Keihanian F, Morovatdar N. Association of polymorphisms of renin angiotensin system and endothelial nitric oxide synthase genes with premature cardiovascular disease in an Iranian population. BMC Cardiovasc Disord. 2023;23(1):254. doi: 10.1186/s12872-023-03276-x.

S76. Park HY, Kwon HM, Kim D, Jang Y, Shim WH, Cho SY, Kim HS. The angiotensin converting enzyme genetic polymorphism in acute coronary syndrome--ACE polymorphism as a risk factor of acute coronary syndrome. J Korean Med Sci. 1997;12(5):391-7. doi: 10.3346/jkms.1997.12.5.391.

S77. Tran DC, Le LHG, Thai TT, Hoang SV, Do MD, Truong BQ. Association between ACE I/D genetic polymorphism and the severity of coronary artery disease in Vietnamese patients with acute myocardial infarction. Front Cardiovasc Med. 2023;10:1091612. doi: 10.3389/fcvm.2023.1091612.

S78. Ferrari M, Mudra H, Grip L, Voudris V, Schächinger V, de Jaegere P, Rieber J, Hausmann D, Rothman M, Koschyk DH, Figulla HR; OPTICUS ACE Substudy. Angiotensin-converting enzyme insertion/deletion polymorphism does not influence the restenosis rate after coronary stent implantation. Cardiology. 2002;97(1):29-36. doi: 10.1159/000047416.

S79. Gardemann A, Fink M, Stricker J, Nguyen QD, Humme J, Katz N, Tillmanns H, Hehrlein FW, Rau M, Haberbosch W. ACE I/D gene polymorphism: presence of the ACE D allele increases the risk of coronary artery disease in younger individuals. Atherosclerosis. 1998;139(1):153-9. doi: 10.1016/s0021-9150(98)00040-9.

S80. Karayannis G, Tsezou A, Giannatou E, Papanikolaou V, Giamouzis G, Triposkiadis F. Polymorphisms of renin-angiotensin system and natriuretic peptide receptor A genes in patients of Greek origin with a history of myocardial infarction. Angiology. 2010;61(8):737-43. doi: 10.1177/0003319710373091.

S81. Koch W, Kastrati A, Mehilli J, Böttiger C, von Beckerath N, Schömig A. Insertion/deletion polymorphism of the angiotensin I-converting enzyme gene is not associated with restenosis after coronary stent placement. Circulation. 2000;102(2):197-202. doi: 10.1161/01.cir.102.2.197.

S82. Nakauchi Y, Suehiro T, Yamamoto M, Yasuoka N, Arii K, Kumon Y, Hamashige N, Hashimoto K. Significance of angiotensin I-converting enzyme and angiotensin II type 1 receptor gene polymorphisms as risk factors for coronary heart disease. Atherosclerosis. 1996;125(2):161-9. doi: 10.1016/0021-9150(96)05866-2.

S83. Narne P, Ponnaluri KC, Singh S, Siraj M, Ishaq M. Relationship between angiotensin-converting enzyme gene insertion/deletion polymorphism, angiographically defined coronary artery disease and myocardial infarction in patients with type 2 diabetes mellitus. J Renin Angiotensin Aldosterone Syst. 2012;13(4):478-86. doi: 10.1177/1470320312448947.

S84. Niemiec P, Zak I, Wita K. The D allele of angiotensin I-converting enzyme gene insertion/deletion polymorphism is associated with the severity of atherosclerosis. Clin Chem Lab Med. 2008;46(4):446-52. doi: 10.1515/CCLM.2008.101.

S85. Qiu C, Han Z, Lu W. Association of polymorphisms in angiotensin-converting enzyme and type 1 angiotensin II receptor genes with coronary heart disease and the severity of coronary artery stenosis. J Huazhong Univ Sci Technolog Med Sci. 2007;27(6):660-3. doi: 10.1007/s11596-007-0610-3.

S86. Vaisi-Raygani A, Rahimi Z, Tavilani H, Vaisi-Raygani H, Kiani A, Aminian M, Shakiba E, Shakiba Y, Pourmotabbed T. Synergism between paraoxonase Arg 192 and the angiotensin converting enzyme D allele is associated with severity of coronary artery disease. Mol Biol Rep. 2012;39(3):2723-31. doi: 10.1007/s11033-011-1027-4.

S87. Sigusch HH, Vogt S, Gruber U, Reinhardt D, Lang K, Surber R, Farker K, Müller S, Hoffmann A. Angiotensin-I-converting enzyme DD genotype is a risk factor of coronary artery disease. Scand J Clin Lab Invest. 1997;57(2):127-32. doi: 10.1080/00365519709056380.

S88. Wang XL, McCredie RM, Wilcken DE. Genotype distribution of angiotensin-converting enzyme polymorphism in Australian healthy and coronary populations and relevance to myocardial infarction and coronary artery disease. Arterioscler Thromb Vasc Biol. 1996;16(1):115-9. doi: 10.1161/01.atv.16.1.115.

S89. Pfohl M, Athanasiadis A, Koch M, Clemens P, Benda N, Häring HU, Karsch KR. Insertion/deletion polymorphism of the angiotensin I-converting enzyme gene is associated with coronary artery plaque calcification as assessed by intravascular ultrasound. J Am Coll Cardiol. 1998;31(5):987-91. doi: 10.1016/s0735-1097(98)00044-8.

S90. Dzimiri N, Basco C, Moorji A, Meyer BF. Angiotensin-converting enzyme polymorphism and the risk of coronary heart disease in the Saudi male population. Arch Pathol Lab Med. 2000;124(4):531-4. doi: 10.5858/2000-124-0531-ACEPAT

S91. Pfohl M, Koch M, Prescod S, Haase KK, Häring HU, Karsch KR. Angiotensin I-converting enzyme gene polymorphism, coronary artery disease and myocardial infarction. An angiographically controlled study. Eur Heart J. 1999;20(18):1318-25. doi: 10.1053/euhj.1999.1543.
